# Structural Revision of the C_16_ Sesquiterpene Hegelenether and the Mechanism of C6‐Methylation in Terpene Biosynthesis

**DOI:** 10.1002/anie.202525672

**Published:** 2026-01-15

**Authors:** Heng Li, Gregor Schnakenburg, Michael Groll, Jeroen S. Dickschat

**Affiliations:** ^1^ Kekulé Institute of Organic Chemistry and Biochemistry University of Bonn Gerhard‐Domagk‐Straße 1 53121 Bonn Germany; ^2^ Institute of Inorganic Chemistry University of Bonn Gerhard‐Domagk‐Straße 1 53121 Bonn Germany; ^3^ Center for Protein Assemblies Department Bioscience School of Natural Sciences Technical University Munich Ernst‐Otto‐Fischer‐Straße 8 85748 Garching Germany

**Keywords:** Isotopic labeling, Marxdiol, Stereochemistry, Terpene methyltransferases, X‐ray crystallography

## Abstract

Non‐canonical methylation events generate terpene structures that evade classical biosynthetic predictions, as exemplified by the proposed C_16_ terpene hegelenether. Here, we show that this natural product is misassigned and revise its structure to the dihydroxylated sesquiterpenoid marxdiol. Its absolute configuration and that of its precursor prekantenol pyrophosphate were determined through terpene synthase‐mediated incorporation of stereoselectively labeled probes. To explain the initiating C6 methylation, we solved the crystal structure of the methyltransferase C6‐FPP‐MT with SAH and FPP, revealing a compact aromatic pocket that enforces *Si*‐face methylation and Glu165‐mediated deprotonation. These insights define how the active site controls regio‐ and stereochemistry and provide a structural basis for identifying related methyl‐modified terpenes in uncharacterized biosynthetic pathways.

## Graphical abstract

The structure of the non‐canonical C_16_ sesquiterpene named hegelenether was revised to the structure of marxdiol, and its absolute configuration was assigned. Isotopic labeling experiments revealed the stereochemical course of the methyltransferase involved in its biosynthesis. The enzyme mechanism was further addressed through protein X‐ray crystallography and structure‐based site‐directed mutagenesis.

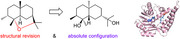

Terpenes represent the largest and structurally most diverse class of natural products, yet their biosynthesis is usually governed by predictable and highly conserved chemical rules. Considering this remarkable diversity, their formation surprisingly relies on a simple logic: only two C_5_ precursors, dimethylallyl pyrophosphate (DMAPP) and isopentenyl pyrophosphate (IPP),^[^ ^1^ ^]^ are sequentially coupled to yield geranyl pyrophosphate (GPP, C_10_), farnesyl pyrophosphate (FPP, C_15_), and higher oligoprenyl pyrophosphates.^[^ ^2^ ^]^ These intermediates are then cyclized by terpene synthases (TSs) through highly complex cationic cascade reactions.

Most terpenes follow the classical isoprene rule first recognized by Wallach and Ruzicka,^[^ ^3^, ^4^ ^]^ but recent research has uncovered deviations that give rise to “non‐canonical” terpenes.^[^ ^5^ ^]^ For instance, geosmin^[^ ^6^ ^]^ (**1**, Scheme 1a) is composed of twelve carbons, explainable by a loss of a C_3_ unit from FPP during terpene cyclization.^[^ ^7^, ^8^ ^]^ In contrast, 2‐methylisoborneol^[^ ^9^ ^]^ (**2**, C_11_) is formed through the action of an S‐adenosylmethionine (SAM)‐dependent methyltransferase (C2‐GPP‐MT) that methylates GPP at C2 to yield 2‐methyl‐GPP, which is then converted into the final product by a terpene synthase.^[^ ^10^, ^11^, ^12^ ^]^ Alternatively, FPP can be methylated at C10 to give α‐presodorifen pyrophosphate (α‐PSPP), which undergoes further cyclization to homosesquiterpenes such as sodorifen (**3**) and anaximandrene (**4**).^[^ ^13^, ^14^, ^15^, ^16^, ^17^, ^18^ ^]^ Two sequential methylation steps of FPP via γ‐PSPP to α‐prechlororaphen pyrophosphate (α‐PCPP) and terpene cyclization account for the biosynthesis of chlororaphens A (**5**) and B (**6**, C_17_).^[^ ^19^, ^20^ ^]^ Together, these pathways demonstrate that methyltransferases (MTs) can generate non‐canonical terpene scaffolds by redefining the size and reactivity of prenyl precursors. Besides, FPP can also be methylated at C6 by C6‐FPP‐MT to yield prekantenol pyrophosphate (PKPP, Scheme 1b), which serves as the precursor to eudesmane‐type homosesquiterpenes: SmoTC from *Streptomyces morookaense* DSM 40 503 converts PKPP into jaegerol (**7**), whereas the reported products of SluTC from *Streptomyces lunaelactis* DSM 42 149 include *epi*‐weberol (**8**) and hegelenether (**9**).^[^ ^21^ ^]^ In this work, we revise the structure of **9**, define its absolute configuration, and elucidate its biosynthesis by combining isotopic labeling with structural and mutational analysis of C6‐FPP‐MT.

**Scheme 1:**
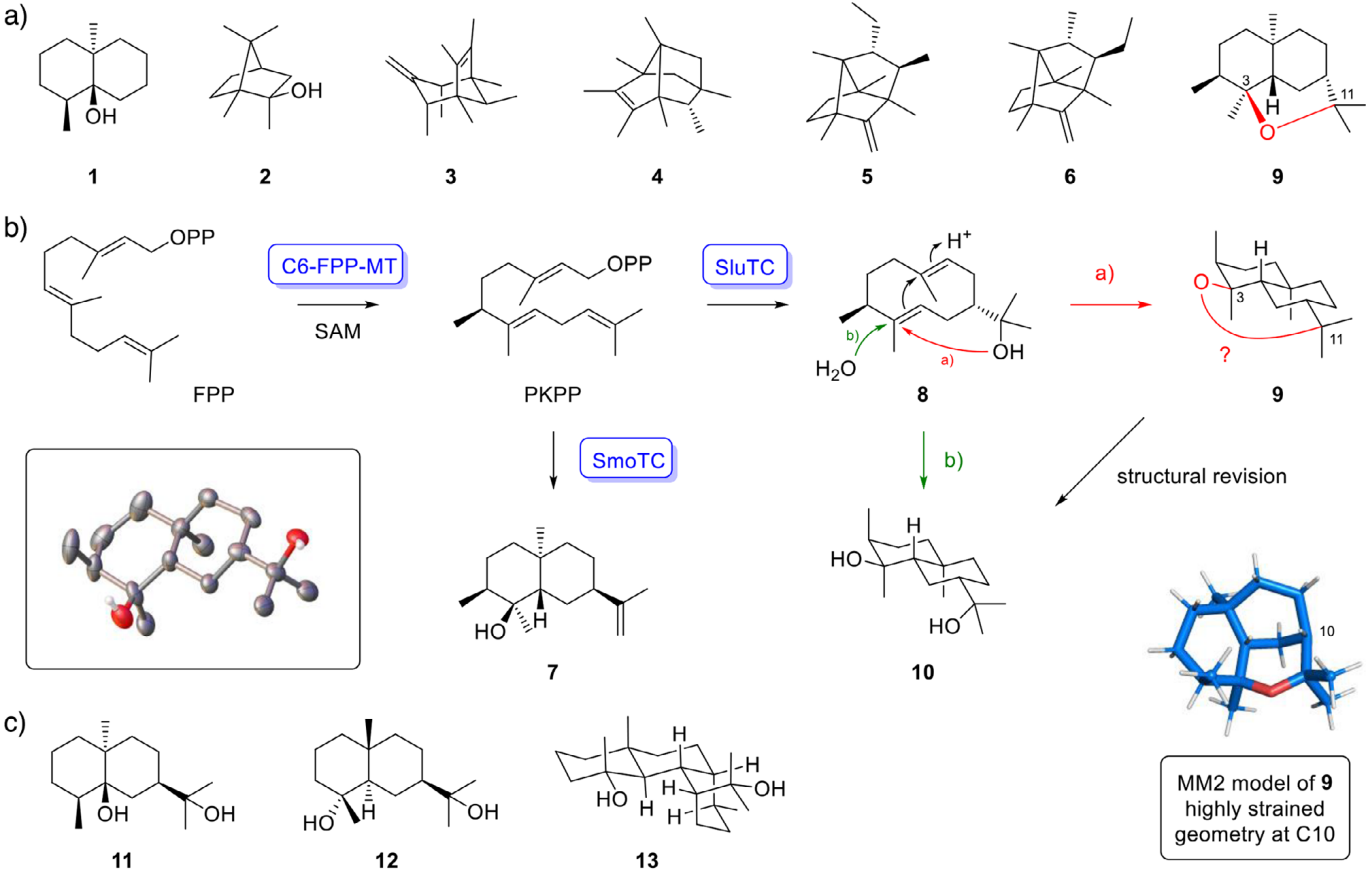
Non‐canonical terpenes. a) Structures of geosmin (**1**), the homoterpenes **2**–**6**, and the previously proposed structure of hegelenether (**9**). b) Formation of PKPP by C6‐FPP‐MT, followed by cyclization to jaegerol (**7**) by SmoTC, or to *epi*‐weberol (**8**) and the reported compound **9** by SluTC.^[^ ^21^ ^]^ The proposed ether bridge in **9** (highlighted in red) is sterically impossible, as illustrated by the computed structure (blue). The revised structure marxdiol (**10**) results from the terminal attack of water during the cyclization of **8**. Box: ORTEP representation of the X‐ray structure of **10**. c) Known terpene diols produced by TSs.

During our work on SmoTC, which revealed its activity with FPP as a germacrene A synthase,^[^ ^22^ ^]^ several TSs from the same phylogenetic branch within a tree of 5677 homologs (Figure S1) had been investigated by Barra and coworkers.^[^ ^21^ ^]^ In each case, the TS gene was accompanied by an MT gene, whose product catalyzes FPP methylation at C6 to form PKPP. Subsequently, the genetically clustered TSs convert PKPP into eudesmane‐type homosesquiterpenes. Among the reported products, **9** attracted our attention, because their proposed structure appeared sterically impossible. Specifically, the distance between C3 and C11 is too long to be bridged by a single ether oxygen (Scheme 1), a conclusion supported by a structure computed using the MM2 molecular mechanics force field, in which all four substituents at C10 point into the same hemisphere. Additional analytical data reinforce these concerns: First, the GC retention index of tricyclic **9** (*I* = 1895)^[^ ^21^ ^]^ is significantly higher than those of the structurally related tricyclic corvol ethers A (*I* = 1494) and B (*I* = 1428).^[^ ^23^ ^]^ The additional carbon in **9** cannot account for a difference of more than 400 index points. Second, the reported IR spectrum shows a broad absorption around 3400 cm^−1 [^ ^21^ ^]^ characteristic of alcohols rather than of ethers (Figure S2). Together, these observations strongly indicate that **9** is not an ether, but a dihydroxylated homosesquiterpene. Furthermore, the original report misinterpreted low‐resolution MS data, claiming a fragment ion at *m*/*z* 236 as a molecular ion and overlooking a fragment ion at *m*/*z* 239 that points to a molecular ion at *m*/*z* 254 (the two mentioned fragment ions arise by loss of a Me group or of water), while high‐resolution MS data were not reported.^[^ ^21^ ^]^ Based on NMR data (Figures S3–S9, Table S1) it is difficult to distinguish between a diol and an ether (the oxygenated carbons are both quaternary and thus a hypothetical ether function cannot be established by ^3^ *J* HMBC correlations). However, using mild chemical ionization (APCI) we have now confirmed the diol nature of the enzyme product (Figure S10), which we designate as marxdiol (**10**, Scheme 1b). Its formation can be rationalized by a second cyclization of **8**, followed by quenching of the terminal carbocation with water instead of intramolecular nucleophilic attack of the hydroxy group. Marxdiol thus, expands the emerging group of terpene diols formed directly by TSs, which currently includes eudesmane‐2,11‐diol (**11**) produced by ZmEDS from *Zea mays*,^[^ ^24^ ^]^ cryptomeridiol (**12**) generated by TwCS from *Tripterygium wilfordii*,^[^ ^25^ ^]^ and jiangning‐3,15‐diol (**13**) formed by CjJS from *Chitinophaga jiangningensis* (Figure 1c).^[^ ^26^ ^]^

**Figure 1:**
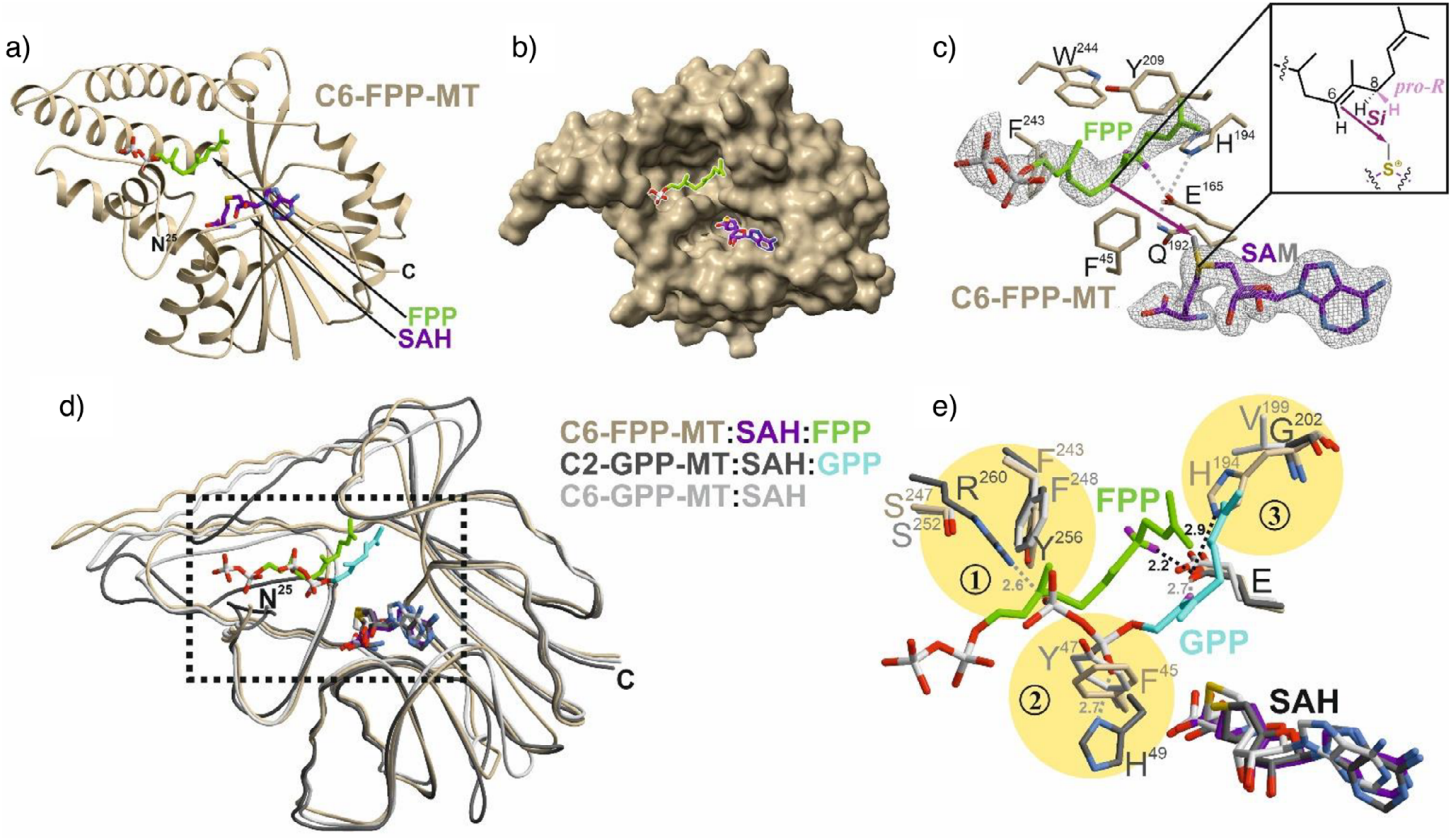
Structural and mechanistic features of C6‐FPP‐MT. a) Ribbon representation of C6‐FPP‐MT from *S. varsoviensis* (PDB ID: 9TB4) complexed with SAH (purple) and FPP (green). b) Molecular surface highlighting the open active site (residues 207–209 omitted to visualize bound FPP). c) Active site with SAH and FPP shown in the 2F_o_‐F_c_ electron‐density map (grey mesh, contoured at 1.0σ; ligands omitted for phasing). Aromatic residues flank C6═C7, positioning C6 toward the modeled Me group of SAM (grey). Glu165 forms a network with His194, aligning the 8‐*pro*‐*R* hydrogen for deprotonation (2.2 Å). The inset illustrates the orientation of the C6═C7 double bond relative to the modeled SAM methyl group, showing that only the *Si*‐face is accessible for methyl transfer. d) Superposition of C6‐FPP‐MT with C2‐GPP‐MT:SAH:GPP (PDB ID: 3VC2) and C6‐GPP‐MT:SAH (PDB ID: 7FBH). e) Close‐up view comparing substrate binding in C6‐FPP‐MT, C2‐GPP‐MT, and C6‐GPP‐MT. The catalytic glutamate (E) adopts a similar orientation in all enzymes, whereas substrate displacement reflects distinct pocket geometries. Three regions impose steric constraints: (1) Tyr256 and Arg260 of C2‐GPP‐MT, which anchor the α‐phosphate of GPP, would clash with the C2═C3 region of FPP in C6‐FPP‐MT; (2) His49 of C2‐GPP‐MT stabilizes the β‐phosphate of GPP, while Phe45 (C6‐FPP‐MT) and Tyr47 (C6‐GPP‐MT) occupy this site; (3) His194 of C6‐FPP‐MT, hydrogen‐bonded to Glu165, would clash with the modeled position of the terminal isoprene unit of GPP in C2‐GPP‐MT. Distances are shown in Å.

The absolute configurations of terpenes can be resolved by using stereoselectively deuterated precursors in which one hydrogen atom of the CH_2_ groups is replaced by deuterium.^[^ ^27^ ^]^ Upon enzymatic conversion by TSs, these precursors introduce artificial stereogenic centers of known configuration into the products, which allows configurational assignment through NOESY‐based relative configuration analysis. For the labeling experiments, DMAPP and (*E*)‐ or (*Z*)‐(4–^13^C,4–^2^H)IPP^[^ ^28^ ^]^ were converted into **10** by *Streptomyces coelicolor* A3(2) FPP synthase (FPPS),^[^ ^29^ ^]^ C6‐FPP‐MT, and SluTC (Scheme 2a). Additional assays were performed using (*R*)‐ and (*S*)‐(1–^13^C,1–^2^H)IPP^[^ ^30^ ^]^ together with *Escherichia coli* isopentenyl diphosphate isomerase (IDI),^[^ ^31^ ^]^ followed by FPPS and SluTC (Scheme 2b). The concomitant ^13^C‐labels enabled highly sensitive HSQC analysis of protium versus deuterium incorporation in CH_2_ groups (Figure S11). Interpretation of the resulting labeling patterns, in light of the stereochemical course of FPP biosynthesis by Cornforth,^[^ ^32^ ^]^ combined with NOESY correlations (Figure S12), established the absolute configuration of **10** (Scheme 2). The same experiments provided configurational assignments for **8** (Figures S13 and S14), and allowed the absolute configuration of PKPP to be deduced from those of **8** and **10**. Additionally, the structure of **10** was confirmed by X‐ray crystallography (Scheme 1b, Table S2), with the restriction that assignment of the absolute configuration through this method remained tentative.

**Scheme 2:**
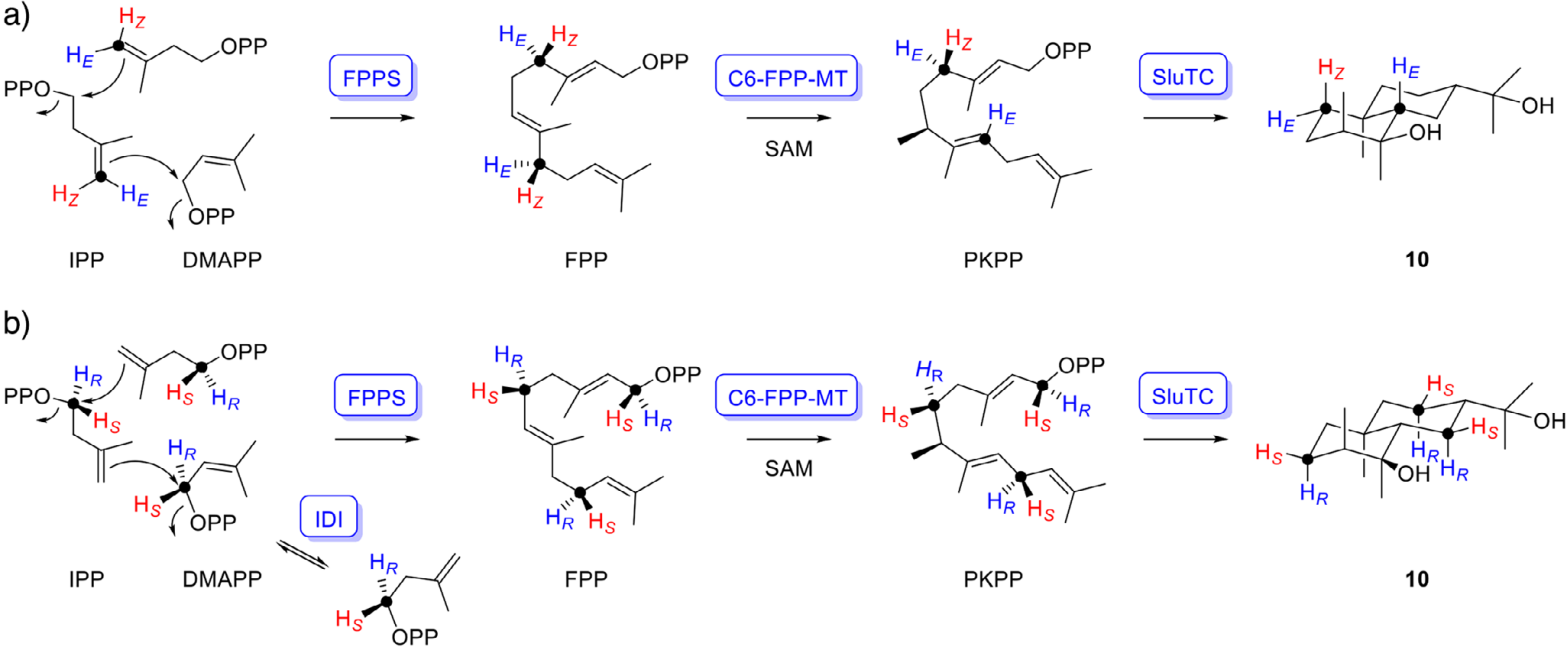
The absolute configuration of **10**. a) Conversion of DMAPP and (*E*)‐ or (*Z*)‐(4–^13^C,4–^2^H)IPP with FPPS, C6‐FPP‐MT, and SluTC into labeled **10**. b) Conversion of (*R*)‐ or *S*)‐(1–^13^C,1–^2^H)IPP with IDI, FPPS, C6‐FPP‐MT, and SluTC into labeled **10**.

Stereochemical insights from the labeling studies, produced by the sequential actions of C6‐FPP‐MT and SluTC, prompted us to structurally analyze C6‐FPP‐MT. The reaction proceeds through CH_3_^+^ transfer from SAM to C6 of FPP, followed by selective removal of the 8‐*pro*‐*R* hydrogen (identical to 4H*_Z_* of IPP; Scheme 2a and Figure S11). The AlphaFold model^[^ ^33^ ^]^ of C6‐FPP‐MT indicated that the first 15 residues form an unstructured segment that does not contact the Rossmann core or the active site. Guided by this prediction, we generated a deletion mutant lacking residue 1 to 15 (C6‐FPP‐MT^Δ15N^). This variant retained full activity (Figure 1b), suggesting that the N terminus is not required for catalysis as proposed for related MTs.^[^ ^34^, ^35^ ^]^ To further dissect the molecular basis of this stereochemical outcome, we crystallized the enzyme from *S. varsoviensis* (Table S4, PDB ID: 9TB4), yielding one complex containing SAH in an unoccupied active site (chain B) and another with bound SAH and FPP (chain A; Figure 1a). Although AlphaFold predicted disorder only for the first 15 amino acids, the crystal structures did not resolve the first 25 residues, showing that this extended segment is flexible and dispensable for proper active‐site organization. The remaining folded portion of C6‐FPP‐MT adopts a canonical Rossmann fold typical of class I SAM‐dependent MTs, featuring a central seven‐stranded parallel β‐sheet flanked by α‐helices. Structural comparison revealed close similarity to C6‐GPP‐MT (BezA) from *S. varsoviensis* (39% sequence identity; RMSD 1.1 Å for 244 C^α^ atoms; PDB ID: 7FBO)^[^ ^36^ ^]^ and to C2‐GPP‐MT from *S. coelicolor* (17% identity; RMSD 1.2 Å for 209 C^α^ atoms; PDB ID: 3VC2).^[^ ^37^ ^]^ In all cases, the conserved core aligns well and binds SAH through conserved interactions.

Comparison of the SAH‐only and SAH:FPP complexes revealed no significant side‐chain rearrangements upon ligand binding. The pyrophosphate (PP) group is only weakly coordinated, and C6‐FPP‐MT lacks a Mg^2+^‐binding motif. Instead, the PP group interacts with residues 29–37 of the N‐terminal helix. The FPP scaffold is tightly stabilized by an extended aromatic network that encloses the ligand within a compact hydrophobic cavity (Figure 1c). The head group is positioned by Tyr37 and Phe45, which flank the C2═C3 double bond and restrict its rotational flexibility. The tail segment is anchored by π–π and van der Waals interactions, with the C10═C11 double bond sandwiched between His194 and Tyr209, while Me12 and Me13 are positioned against Ile205 and Thr211. These interactions define the geometry of the substrate pocket and account for its strict preference for FPP.^[^ ^21^ ^]^ Longer isoprenoid chains would exceed the available space, whereas shorter substrates would be insufficiently stabilized. The key role of these flanking residues in substrate binding is demonstrated for the F45A, H194A, H194N and Y209A variants that showed either very low production (F45A: 0.01 ± 0.001%, H194N: 0.04 ± 0.01%, relative to wild‐type production = 100%) or product levels below the limit of detection (<0.01%) (Figure S15).

Catalysis occurs at the C6═C7 double bond. Phe243 and Trp244 position C6 toward the co‐substrate, thereby establishing the reactive geometry (Figure 1c). The bulky residue Trp244 is essential for this alignment, as demonstrated by product levels below the limit of detection by the W244F variant. In addition, Met47 restricts the rotation of the olefinic plane of the middle isoprene unit and maintains the orientation required for stereoselective methyl transfer. However, the methyl group of the modeled SAM is positioned 6.2 Å from C6, substantially longer than the ∼3.5 Å typically observed in catalytically competent MTs.^[^ ^37^ ^]^ This suggests that the complex captures a pre‐reactive state in which additional conformational tightening is required to bring the catalytic centers into alignment. The absence of the SAM sulfonium cation in the observed SAH complex may contribute to this state by stabilizing a more expanded active‐site geometry and preventing full closure of the catalytic pocket. Such an SAH‐bound conformation could facilitate product release, while a SAM‐bound complex may adopt a more compact conformation that reduces the SAM‐substrate distance to a catalytically competent range. Yet, even in this pre‐reactive arrangement, the orientation of the C6═C7 double bond is fixed so that methyl transfer can occur exclusively from the *Si* face. This accounts for the formation of (*S*)‐PKPP and is fully consistent with the determined absolute configuration of **10**. Following this stereoselective methylation, the transient carbocation at C7 is quenched by Glu165 through 8‐*pro*‐*R* deprotonation (2.2 Å), yielding an (*E*)‐configured double bond, again in agreement with the labeling data (Figure S11). Glu165 is further hydrogen‐bonded to His194 (2.9 Å), allowing His194 to stabilize the C10═C11 double bond and thus linking catalysis in the central region to conformational control of the tail unit. In support of this model, the H194A, H194N, E165A, and E165Q variants showed product levels below the limit of detection, demonstrating that both His194 and Glu165 are indispensable for catalysis (Figure S15). Despite possible indirect effects, the combined structural, stereochemical, labeling, and mutational data support Glu165 as the Brønsted base and an active‐site geometry that enforces *Si*‐face methylation at C6.

Comparison with related MTs indicates that SAH adopts an identical binding mode in C6‐FPP‐MT, C2‐GPP‐MT, and C6‐GPP‐MT (Figure 1d), whereas the substrate‐binding pockets differ markedly, consistent with their distinct methylation patterns. While no substrate complex is available for C6‐GPP‐MT, the ligands in C6‐FPP‐MT and C2‐GPP‐MT occupy equivalent tail‐binding regions. In C2‐GPP‐MT, the PP group and Mg^2+^ cofactor are anchored by extensive interactions with Arg34, Val36, Asn37, His49, Tyr51, Glu81, Tyr256, and Arg260 (Figure S16), whereas C6‐FPP‐MT lacks these coordinating residues, which renders the PP group more flexible. The head unit of FPP in C6‐FPP‐MT overlaps with the PP group of GPP in C2‐GPP‐MT, shifting the substrate into a binding mode that generates several steric conflicts. These arise in three discrete regions that together define the distinct substrate geometries (Figure 1e): (1) Tyr256 and Arg260 of C2‐GPP‐MT, which stabilize the α‐phosphate of GPP, would clash with the C2═C3 region of FPP in C6‐FPP‐MT; (2) His49 of C2‐GPP‐MT coordinates the β‐phosphate of GPP, whereas this pocket is occupied by Phe45 in C6‐FPP‐MT and by Tyr47 in C6‐GPP‐MT; (3) His194 of C6‐FPP‐MT, hydrogen‐bonded to Glu165, would clash with the position of the terminal isoprene unit of GPP in C2‐GPP‐MT. Furthermore, Met47 in C6‐FPP‐MT positions the middle unit of FPP, while the corresponding Tyr51 in C2‐GPP‐MT would interfere with this segment. As a consequence of these differences, the chain of GPP is displaced by ∼4.2 Å toward the cofactor, positioning C2 only 3.2 Å from the SAM methyl group,^[^ ^37^ ^]^ consistent with selective C2‐methylation. In contrast, C6‐FPP‐MT imposes no steric restrictions around the C6═C7 double bond, allowing conformational flexibility that supports C6‐methylation.^[^ ^36^ ^]^

Despite these differences, several key features are conserved across the three MTs. The aromatic residues that stabilize substrate double bonds through π‐stacking are identical in sequence and position: Trp244 in C6‐FPP‐MT is preserved in both C2‐GPP‐MT and C6‐GPP‐MT, and Phe243 is either conserved or substituted by Tyr. The Brønsted base occupies a similar spatial position in all enzymes,^[^ ^36^, ^37^ ^]^ with Glu165 in C6‐FPP‐MT corresponding to Glu173 in C2‐GPP‐MT (3.5 Å from the C2 deprotonation site) and Glu170 in C6‐GPP‐MT. In C6‐FPP‐MT, Glu165 is stabilized by Gln192 and hydrogen‐bonded to His194 (Figures 1e and S17), whereas in C2‐GPP‐MT, Glu173 interacts with Gln84 and Tyr284. Thus, all three enzymes rely on an invariant catalytic glutamate, while differences in the surrounding residues shape distinct substrate‐binding pockets and thereby dictate regioselectivity. In this context, the binding mode of GPP in C6‐GPP‐MT remains speculative and will require experimental validation.

In summary, the structure **9** previously assigned to a C_16_ sesquiterpene named hegelenether is revised to marxdiol (**10**), and its absolute configuration is now firmly established. By combining isotopic labeling, protein X‐ray crystallography of C6‐FPP‐MT, and structure‐based mutagenesis, we obtained the first mechanistic insights into its biosynthesis. These findings rationalize the substrate preference, the site of methylation, and the stereochemical outcome of the reaction. A sequence comparison of 501 MTs closely related to C6‐GPP‐MT reveals a high conservation of active‐site residues (Table S5), indicating that many of these enzymes may share analogous substrate preferences and regio‐ and stereochemical features of methyl transfer. With expanding genome data, our mechanistic framework indicates that the repertoire of methyl‐modified terpenes is far larger than currently known. Mining uncharacterized biosynthetic loci, therefore, offers substantial potential for discovering new structural families and deepening our understanding of non‐canonical terpene biosynthesis.

## Supporting Information

The authors have cited additional references within the Supporting Information.^[^ ^38^, ^39^, ^40^, ^41^, ^42^, ^43^, ^44^, ^45^, ^46^, ^47^, ^48^ ^]^

## Conflict of Interests

The authors declare no conflict of interest.

## Supporting information



Supporting Information

## Data Availability

The data that support the findings of this study are available in the Supporting Information of this article.
