# Supplementary material for: Structural Revision of the C16 Sesquiterpene Hegelenether and the Mechanism of C6‐Methylation in Terpene Biosynthesis
Source: Angew Chem Int Ed Engl. 2026 Jan 15;65(9):e25672. doi: 10.1002/anie.202525672 (PMC12929941; doi:10.1002/anie.202525672)
Supplement: Supplementary file 1 — Supporting Information [file ANIE-65-e25672-s001.pdf]

Table of Contents

Phylogenetic analysis of TC homologs	2
Bioinformatic, microbiological and biochemical methods	3
Analytical and Spectroscopic Methods	4
IR spectrum of **10**	5
Structure elucidation and NMR data of **10**	6
HRMS spectrum (APCI) of **10**	13
The absolute configuration of **10**	14
NOESY correlations of **10**	15
The absolute configuration of **8**	16
X-ray crystallographic analysis of **10**	18
Enzyme crystallography and site-directed mutagenesis	20
Residues involved in Mg^{2+} and substrate binding in C2-GPP-MT	25
The conserved Brønsted base Glu165	26
Conservation of active site residues in C6-FPP-MT homologs	27
References	28

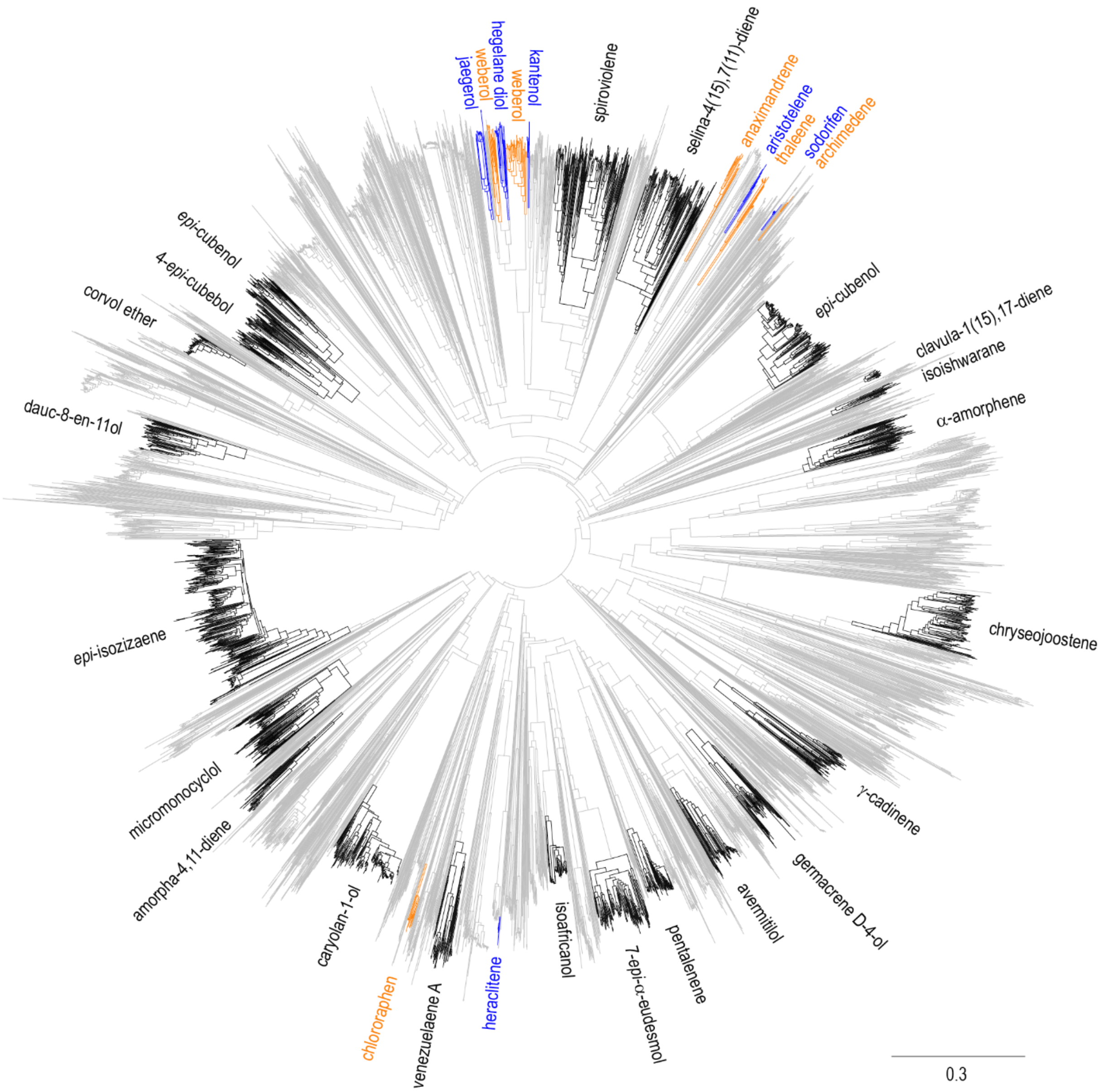

Figure S1. Phylogenetic tree constructed from the amino acid sequences of 5667 bacterial TS homologs. Clades of closely related canonical sesqui- and diterpene synthases with at least one characterised member are shown in black, those of non-canonical C_{16} or C_{17} sesquiterpene synthases are shown in orange and blue. The scale bar represents substitutions per site.

Bioinformatic, microbiological and biochemical methods

Construction of phylogenetic tree

Through continuous data collection over several years, BLAST searches using the amino acid sequences of characterised terpene synthases as probes resulted in the discovery of the amino acid sequences of 5667 bacterial terpene synthase homologs. All sequences included in the phylogenetic tree (Figure S1) were individually inspected for the presence of the highly conserved motifs usually observed in type I terpene synthases. The tree was constructed using the tree builder function of Geneious (alignment type: global alignment with free end gaps, cost matrix: Blosum45, genetic distance model: Jukes-Cantor, tree build method: neighbor-joining, gap open penalty: 8, gap extension penalty: 2).

Gene cloning, expression and protein purification

The genes for C6-FPP-MT (WP_048831493) and for SluTC (WP_108148729) were cloned and expressed in *Escherichia coli* BL21 (DE3) as reported previously.[1] Protein concentrations were measured by Bradford assay,[2] typically showing yields of 3 mg mL^{-1}.

Conversion of FPP with C6-FPP-MT and SluTC and product isolation

FPP (50 mg, 116 μmol) and *S*-adenosyl-L-methionine disulfate tosylate (100 mg, 130 μmol, 1.12 eq) was dissolved in incubation buffer (50 mL), followed by the addition of an enzyme preparation of C6-FPP-MT (6 mL, 3 mg mL^{-1}) and SluTC (9 mL, 3 mg mL^{-1}). The reaction mixture was incubated for 16 h at 30 °C, followed by the extraction with diethyl ether (3x 100 mL). The combined extracts were dried with $MgSO_4$ and the solvent was evaporated. The crude product was purified via column chromatography on silica gel to yield pure **8** (10.0 mg, 42 μmol, 36%) as a colourless oil and **10** (9.0 mg, 35 μmol, 30%) as a colourless solid.

epi-Weberol (**8**). TLC (Et_2O): R_f = 0.74. GC (HP-5MS): *I* = 1785. MS (EI, 70 eV): *m*/*z* (%) = 41 (2), 59 (5), 67 (3), 79 (4), 95 (20), 105 (8), 119 (8), 133 (8), 147 (5), 161 (4), 175 (15), 189 (3), 203 (7), 218 (6), 236 (1). HR-MS (Q-TOF, 70 eV): calc. $[C_{16}H_{28}O]^+$ *m*/*z* = 236.2135; found: *m*/*z* = 236.2130. Optical rotation: $[\alpha]_D^{25}$ = 0.4 (*c* 0.75, CH_2Cl_2). NMR data were identical to literature data.[1]

Marxdiol (**10**). TLC (Et_2O): R_f = 0.13. GC (HP-5MS): *I* = 1918. MS (EI, 70 eV): *m*/*z* (%) = 43 (5), 55 (2), 59 (7), 81 (7), 95 (7), 109 (6), 123 (11), 139 (8), 149 (4), 163 (16), 179 (9), 196 (4), 203 (5), 218 (3), 236 (2), 254 (0.2). HR-MS (APCI): calc. $[C_{16}H_{31}O_2]^+$ *m*/*z* = 255.2319; found: *m*/*z* = 255.2319. Optical rotation: $[\alpha]_D^{25}$ = +42.3 (*c* 0.47, CH_2Cl_2). The IR spectrum (ATR) is shown in Figure S2. NMR data are given in Table S1.

Isotopic labeling experiments

A solution of *S*-adenosyl-L-methionine disulfate tosylate (2 mg) and (*R*)- or (*S*)-(1-^{13}C,1-^{2}H)IPP (1 mg) in aqueous NH_4HCO_3 (1 mL; 25 mM) was added to incubation buffer (2 mL; 50 mM Tris/HCl, 10 mM $MgCl_2$, 10% glycerol, 20 mM β-cyclodextrin, pH = 7.5), followed by the addition of preparations of the purified enzymes FPPS (0.5 mL), IDI (0.5 mL), C6-FPP-MT (0.5 mL) and SluTC (0.5 mL) in elution buffer (enzyme concentrations 3 mg mL^{-1}; 20 mM Na_2HPO_4, 500 mM NaCl, 500 mM imidazole, 1 mM $MgCl_2$, pH = 7.4). Analogous incubation experiments with *S*-adenosyl-L-methionine disulfate tosylate (2 mg), DMAPP (1 mg) and (*E*)- or (*Z*)-(4-^{13}C,4-^{2}H)IPP (1 mg) were performed in the same way. In this case, the IDI enzyme preparation was substituted by an equal volume of incubation buffer. After incubation at 28 °C overnight, the formed products were extracted with C_6D_6 (400 μL + 200 μL) and the extracts were analysed by NMR and GC/MS. The results are shown in Figures S11, S13 and S14.

Analytical and Spectroscopic Methods

GC/MS

GC/MS analyses were performed on a 5977A GC/MSD system (Agilent, Santa Clara, CA, USA) composed of a 7890B GC and a 5977A mass selective detector. The GC was equipped with a HP5-MS fused silica capillary column (30 m, 0.25 mm i. d., 0.50 μm film). Specific GC settings were 1) inlet pressure: 77.1 kPa, He at 23.3 mL min^{-1}, 2) injection volume: 1 μL, 3) temperature program: 5 min at 50 °C increasing at 10 °C min^{-1} to 320 °C, 4) 60 s valve time, and 5) carrier gas: He at 1.2 mL min^{-1}. MS settings were 1) source: 230 °C, 2) transfer line: 250 °C, 3) quadrupole: 150 °C and 4) electron energy: 70 eV. Retention indices (*I*) were determined from retention times in comparison to the retention times of *n*-alkanes (C_7-C_{40}).

HRMS

High resolution mass spectra were recorded on an Orbitrap XL instrument (APCI; Thermo Fisher Scientific, Waltham, MA, USA) or using a 7890B/7200 series gas chromatography/accurate mass Q-ToF detector system (Agilent). The GC was equipped with a HP5-MS fused silica capillary column (30 m, 0.25 mm i. d., 0.50 μm film). GC settings were 1) injection volume: 1 μL, 2) temperature program: 5 min at 50 °C, increasing 10 °C min^{-1} to 320 °C, 3) split ratio: 5:1, 60 s valve time and 4) carrier gas flow: He at 1 mL min^{-1}. MS settings were 1) inlet pressure: 83.2 kPa, He flow at 24.6 mL min^{-1}, 2) transfer line temperature: 250 °C, 3) ionization energy: 70 eV.

NMR spectroscopy

NMR spectra were recorded on a Bruker (Billerica, MA, USA) Avance I (300 MHz), Avance I (400 MHz), Avance I (500 MHz), Avance III HD Prodigy (500 MHz) or an Avance III HD Cryo (700 MHz) NMR spectrometer. Spectra were measured in C_6D_6 and referenced against solvent signals (^{1}H-NMR, residual proton signal: δ = 7.16; ^{13}C-NMR: δ = 128.06).[3]

IR spectroscopy

IR spectra were recorded on a Bruker α infrared spectrometer with a diamond ATR probehead. Peak intensities are given as s (strong), m (medium), w (weak) and br (broad).

Optical rotations

Optical rotations were recorded on a Modular Compact Polarimeter MCP 100 (Anton Paar, Graz, Austria). The temperature setting was 25 °C; the wavelength of the light used was 589 nm (sodium D line); the path-length was 10 cm; the compound concentrations *c* are given in g 100 mL^{-1}.

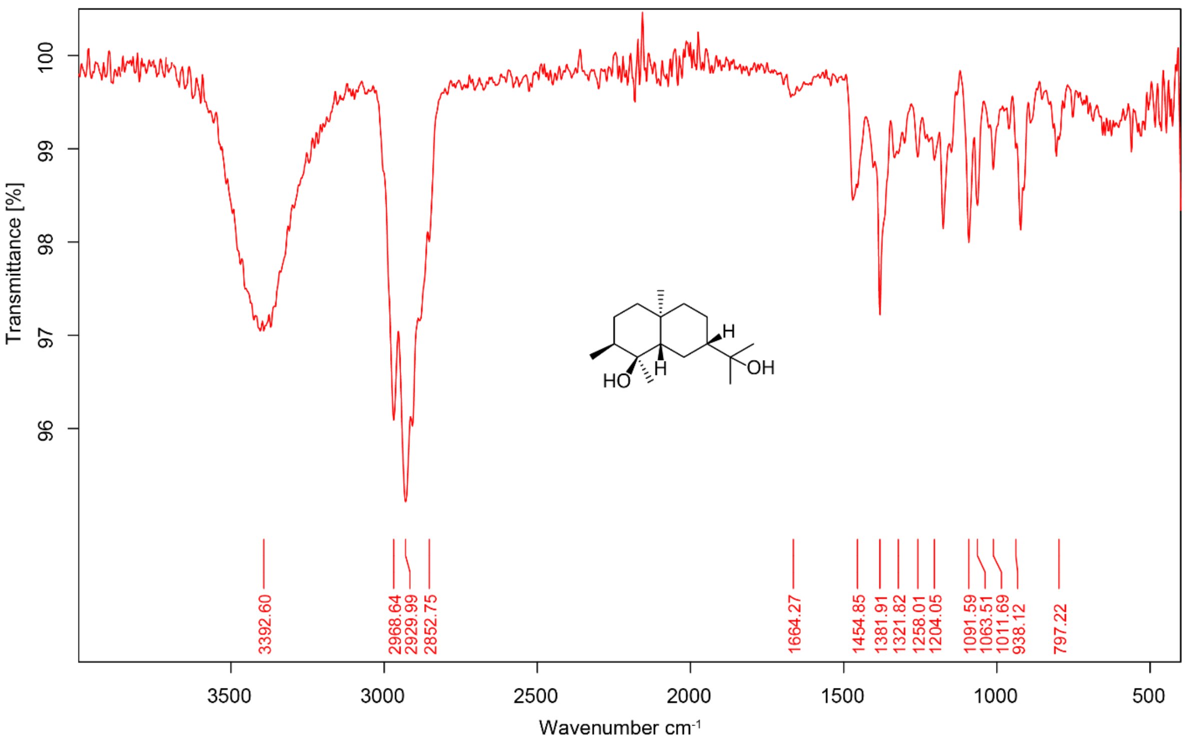

Figure S2. IR spectrum of marxdiol (**10**).

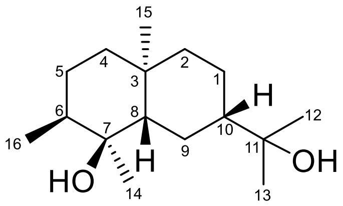

10

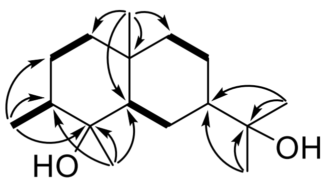

Figure S3. Structure elucidation of marxdiol (**10**). Bold: ^{1}H,^{1}H-COSY, single headed arrows: key HMBC. Carbon numbering follows FPP numbering to indicate the origin of each carbon, and Me16 derives from SAM.

Table S1. NMR data of marxdiol (**10**) in C_6D_6 recorded at 298 K.

C[a]	type	^{13}C[b]	^{1}H[b]
1	CH_2	23.0	1.48 (dm, J = 12.8)
			1.20 (m)
2	CH_2	45.2	1.33 (ddd, J = 12.5, 3.2, 3.2)
			1.07 (m)
3	C_q	34.7	–
4	CH_2	35.0	1.30 (m)
			1.03 (m)
5	CH_2	26.86	1.73 (m)
			1.28 (m)
6	CH	41.2	1.71 (m)
7	C_q	73.1	–
8	CH	48.0	1.36 (dd, J = 12.3, 3.6)
9	CH_2	21.5	1.98 (dm, J = 12.4)
			1.00 (m)
10	CH	50.3	1.23 (m)
11	C_q	72.3	–
12	CH_3	27.9	1.10 (s)
13	CH_3	26.9	1.08 (s)
14	CH_3	25.2	1.07 (s)
15	CH_3	19.0	0.79 (s)
16	CH_3	14.2	1.05 (d, J = 7.3)

[a] Carbon numbering as shown in Figure S3 indicates the origin of each carbon from FPP by same number. [b] Chemical shifts δ in ppm, multiplicity: s = singlet, d = doublet, m = multiplet, coupling constants J are given in Hertz.

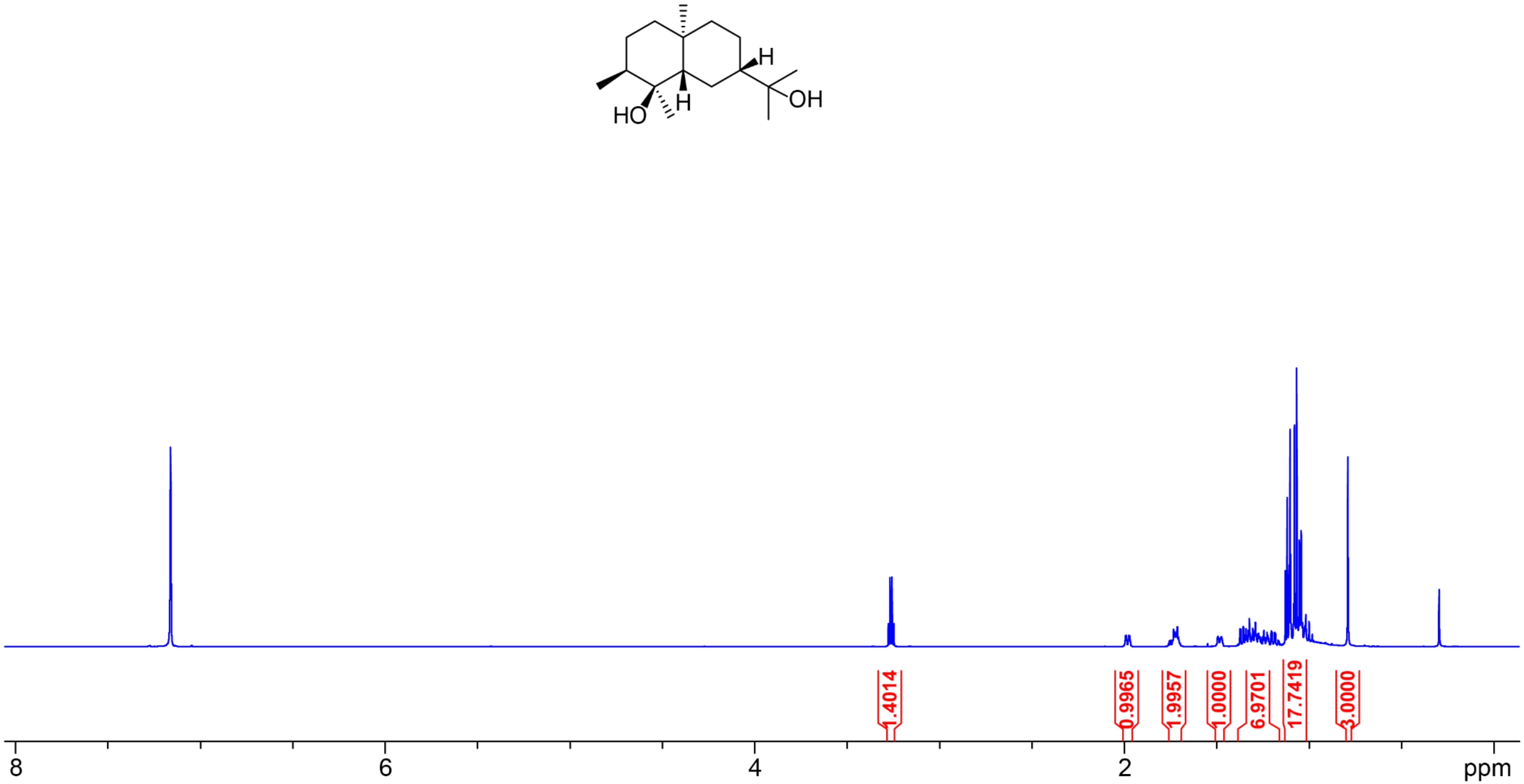

Figure S4. ^{1}H-NMR spectrum of **10** (700 MHz, C_6D_6).

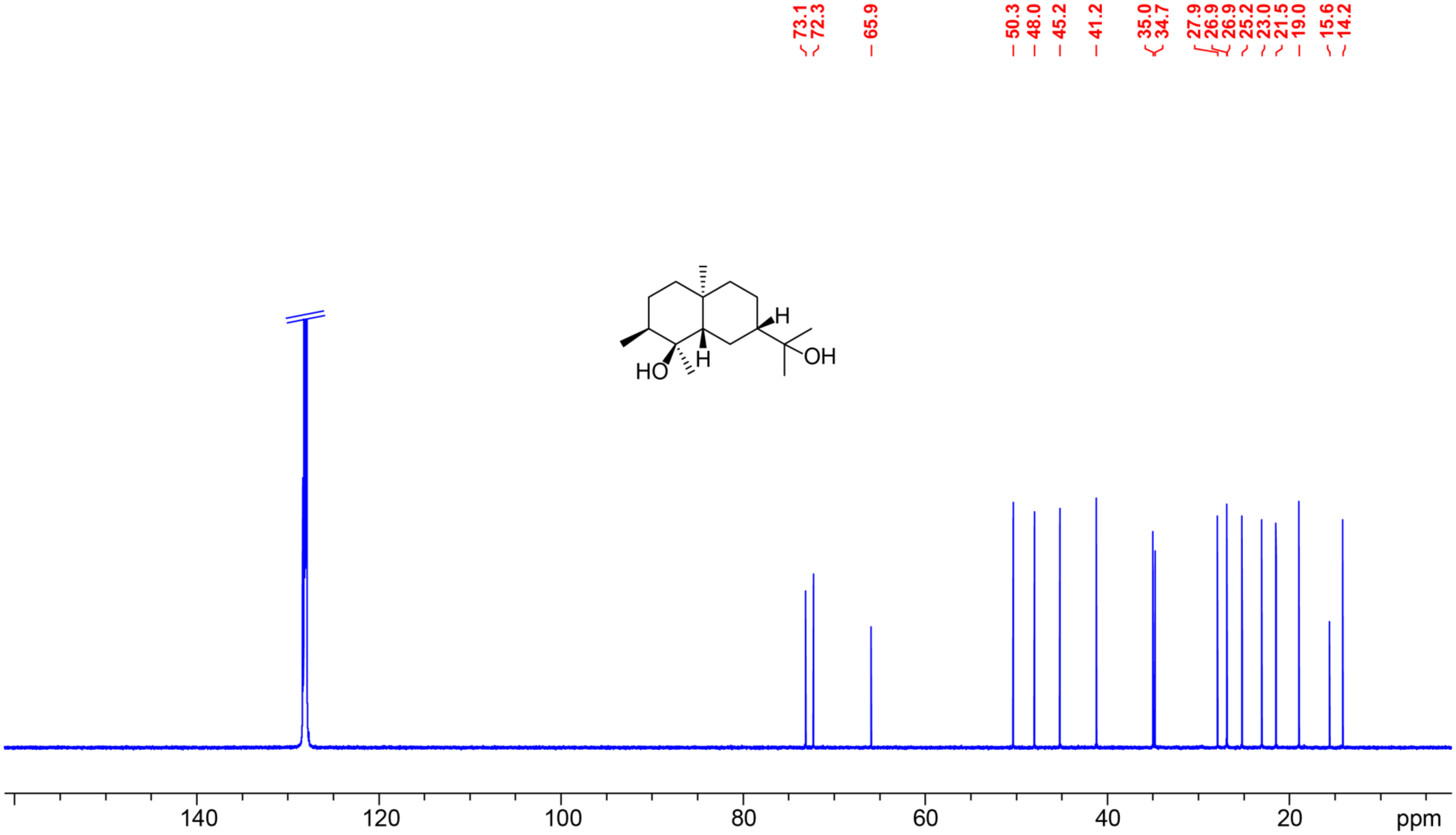

Figure S5. ^{13}C-NMR spectrum of **10** (176 MHz, C_6D_6).

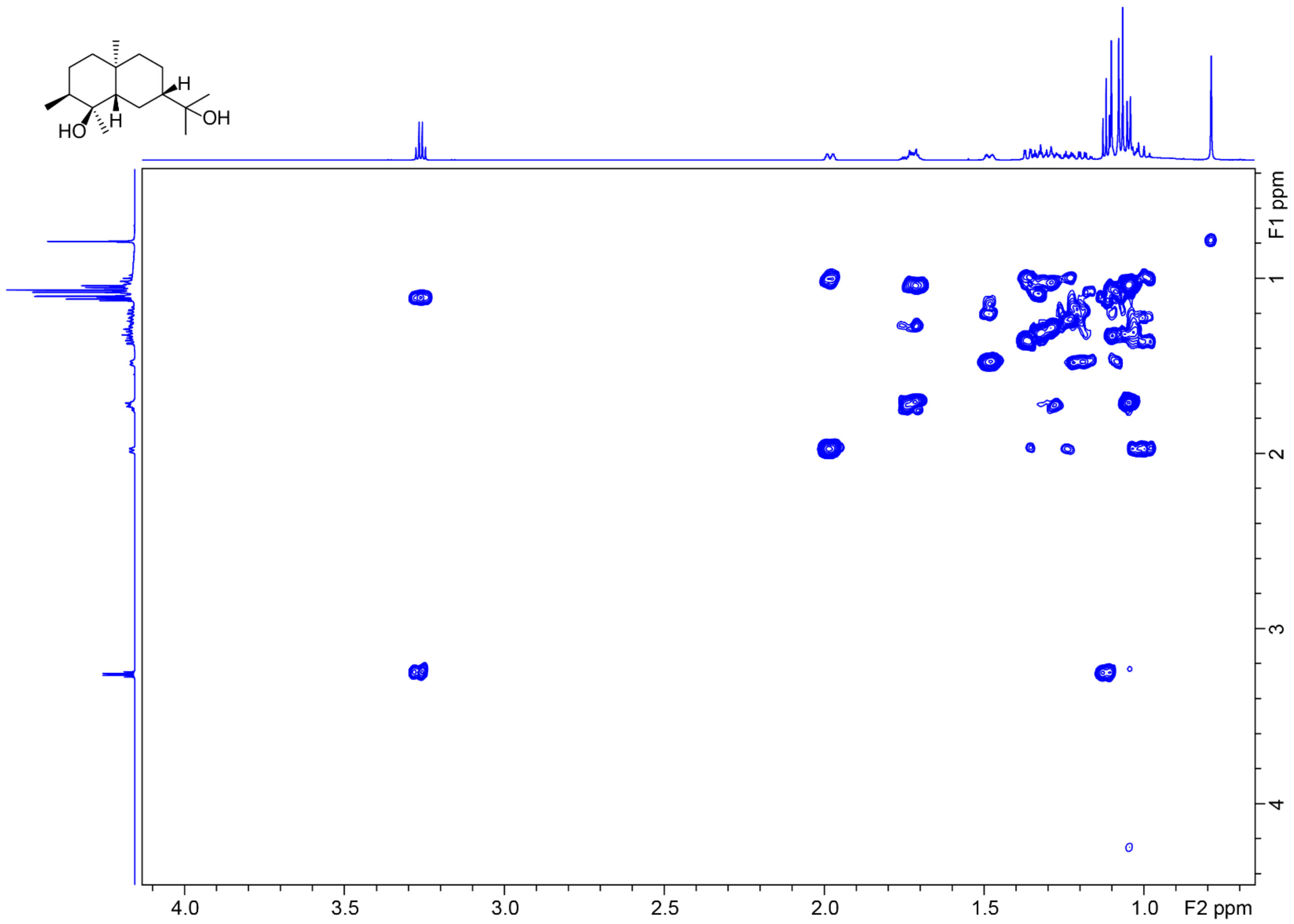

Figure S6. ^{1}H-^{1}H COSY NMR spectrum of **10** (C_6D_6).

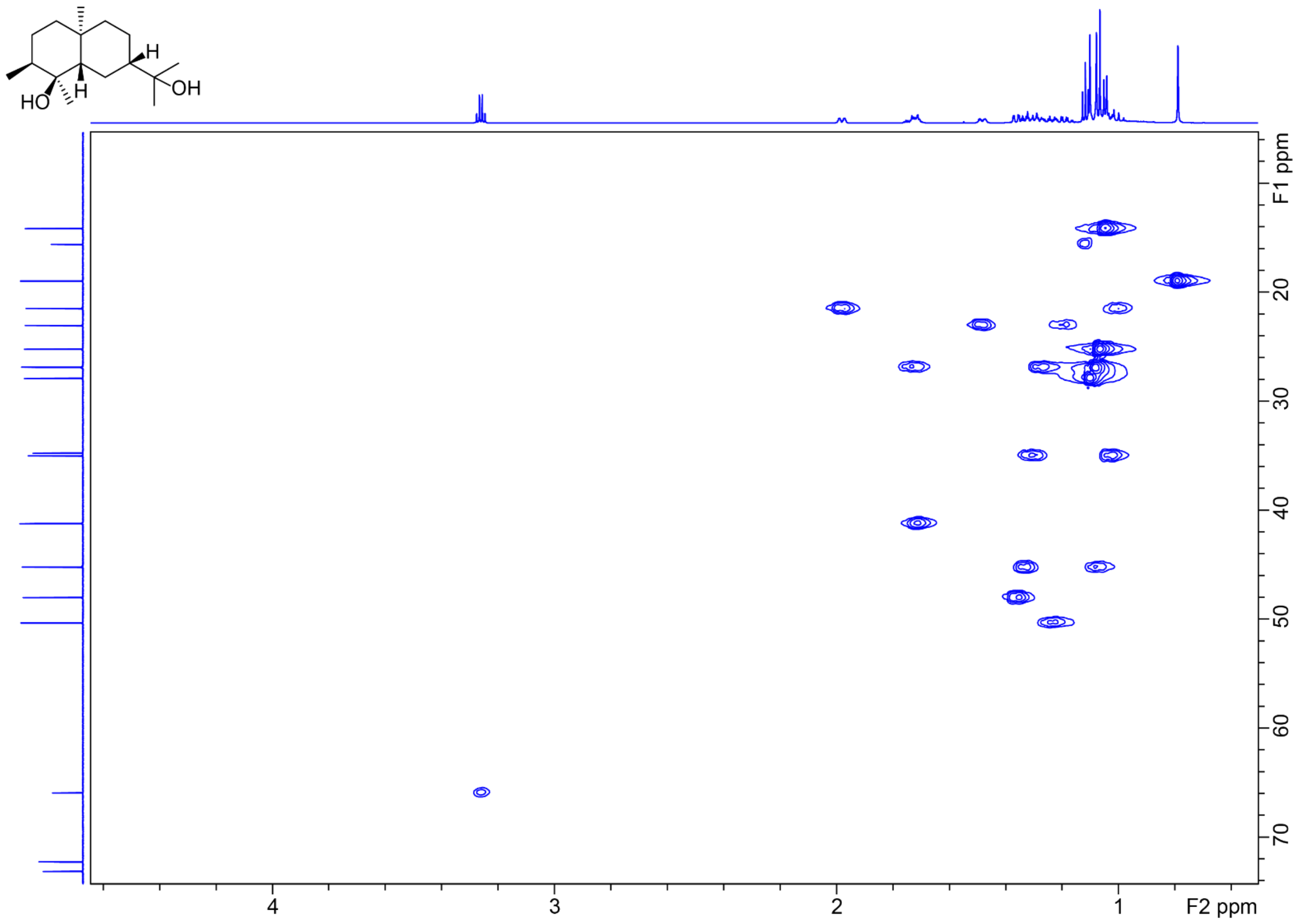

Figure S7. HSQC NMR spectrum of **10** (C_6D_6).

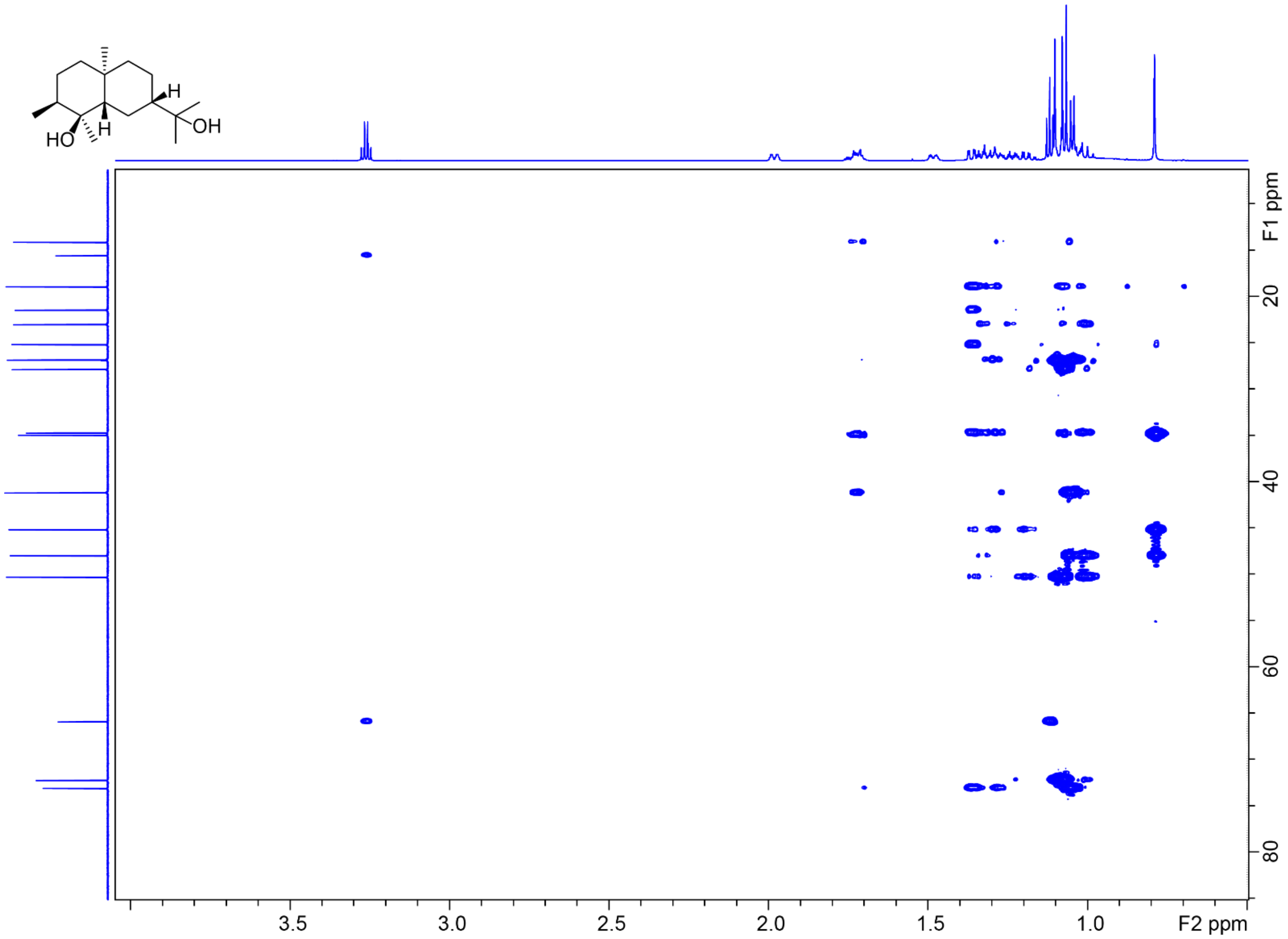

Figure S8. HMBC NMR spectrum of **10** (C_6D_6).

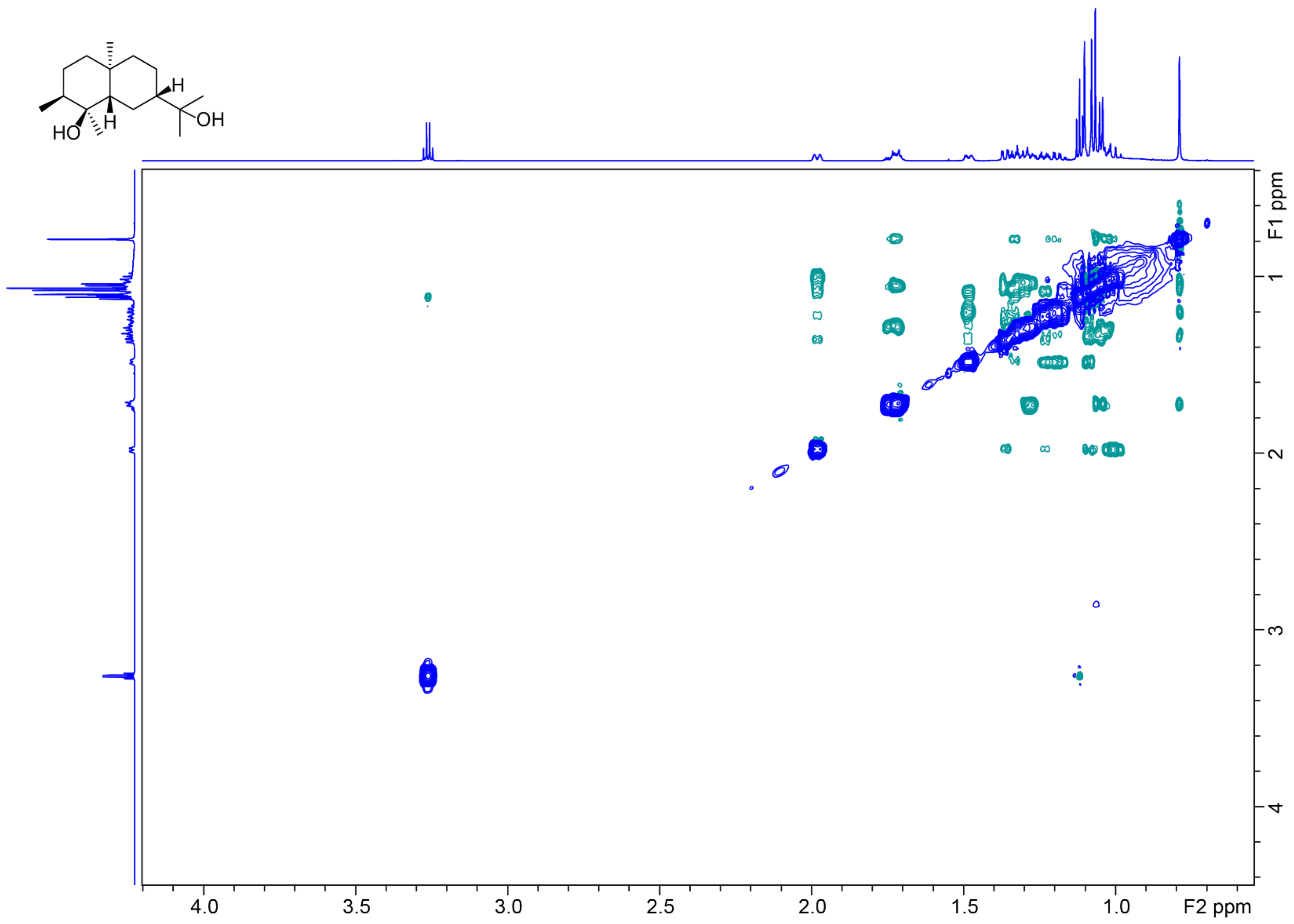

Figure S9. NOESY NMR spectrum of **10** (C_6D_6).

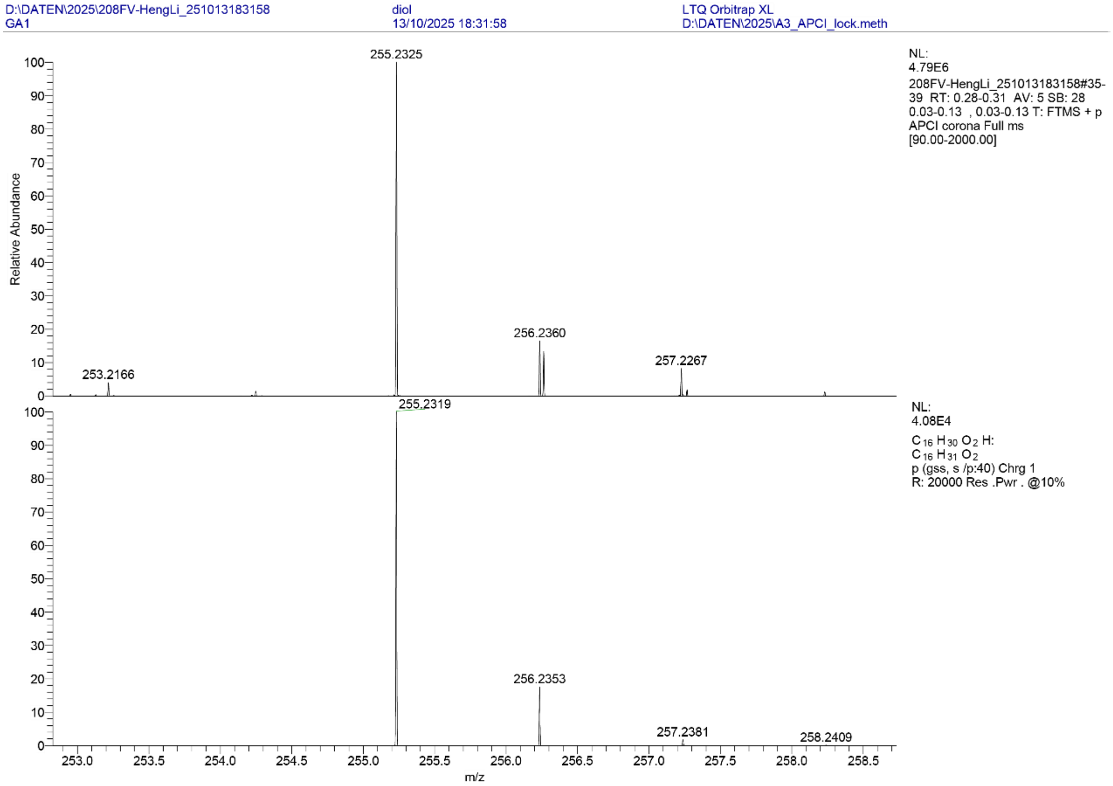

Figure S10. High-resolution mass spectrum (APCI) of **10**. Top: measured mass spectrum, bottom: calculated mass spectrum for $C_{16}H_{31}O_2^+$.

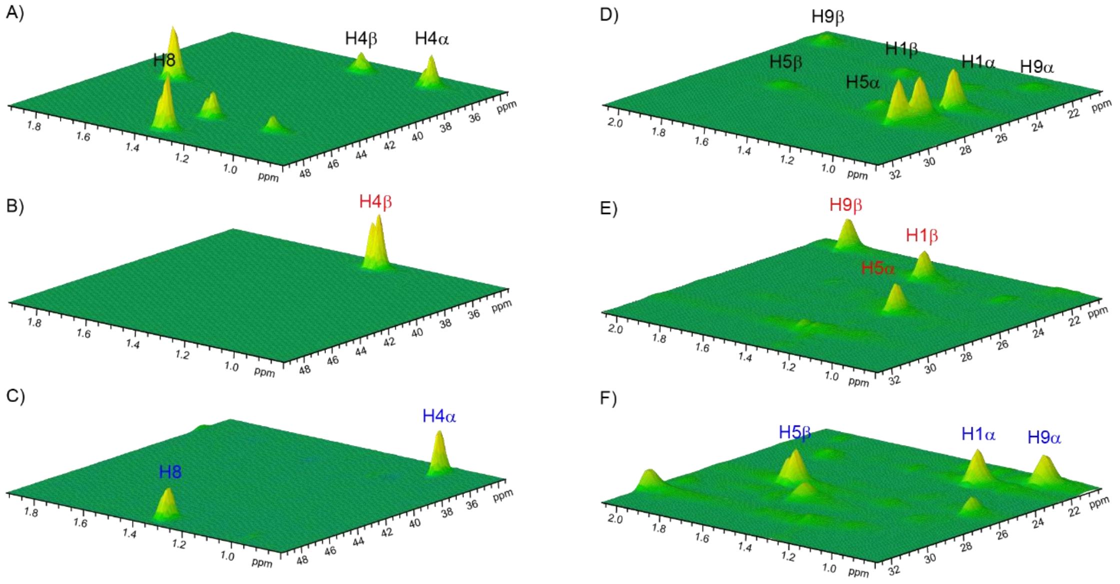

Figure S11. The absolute configuration of **10**. HSQC spectra of A) unlabeled **10** showing the region for C4 and C8, B) labeled **10** obtained from (*E*)-(4-^{13}C,4-^{2}H)IPP (signals for H_Z=^{1}H are observed), and C) labeled **10** obtained from (*Z*)-(4-^{13}C,4-^{2}H)IPP (signals for H_E=^{1}H are observed), D) unlabeled **10** showing the region for C1, C5 and C9, E) labeled **10** obtained from (*R*)-(1-^{13}C,1-^{2}H)IPP (signals for H_S=^{1}H are observed), and F) labeled **10** obtained from (*S*)-(1-^{13}C,1-^{2}H)IPP (signals for H_R=^{1}H are observed). Together with the observed NOESY correlations for **10** (Figure S12) the absolute configuration as shown in Scheme 2 of main text can be assigned. The HSQC spectra in B) and C) additionally confirm the selective loss of the 8-*pro-R* hydrogen (originating from 4H_Z of IPP) in the deprotonation to PKPP during C6-FPP-MT catalysis.

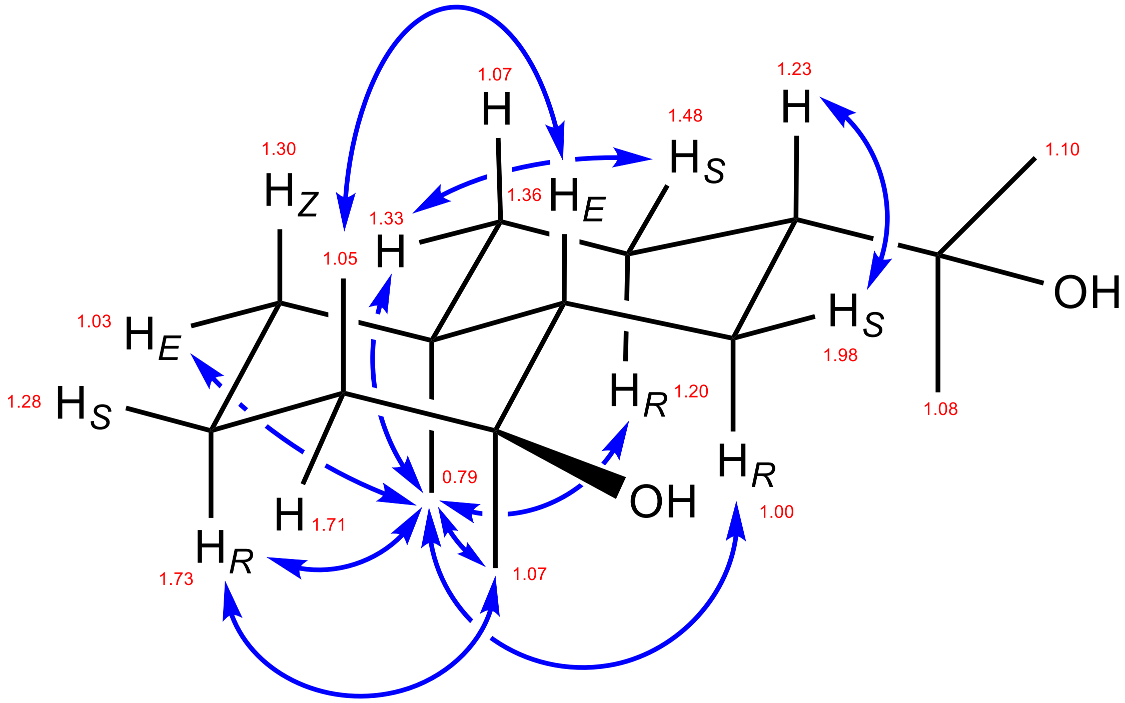

Figure S12. NOESY correlations of **10**.

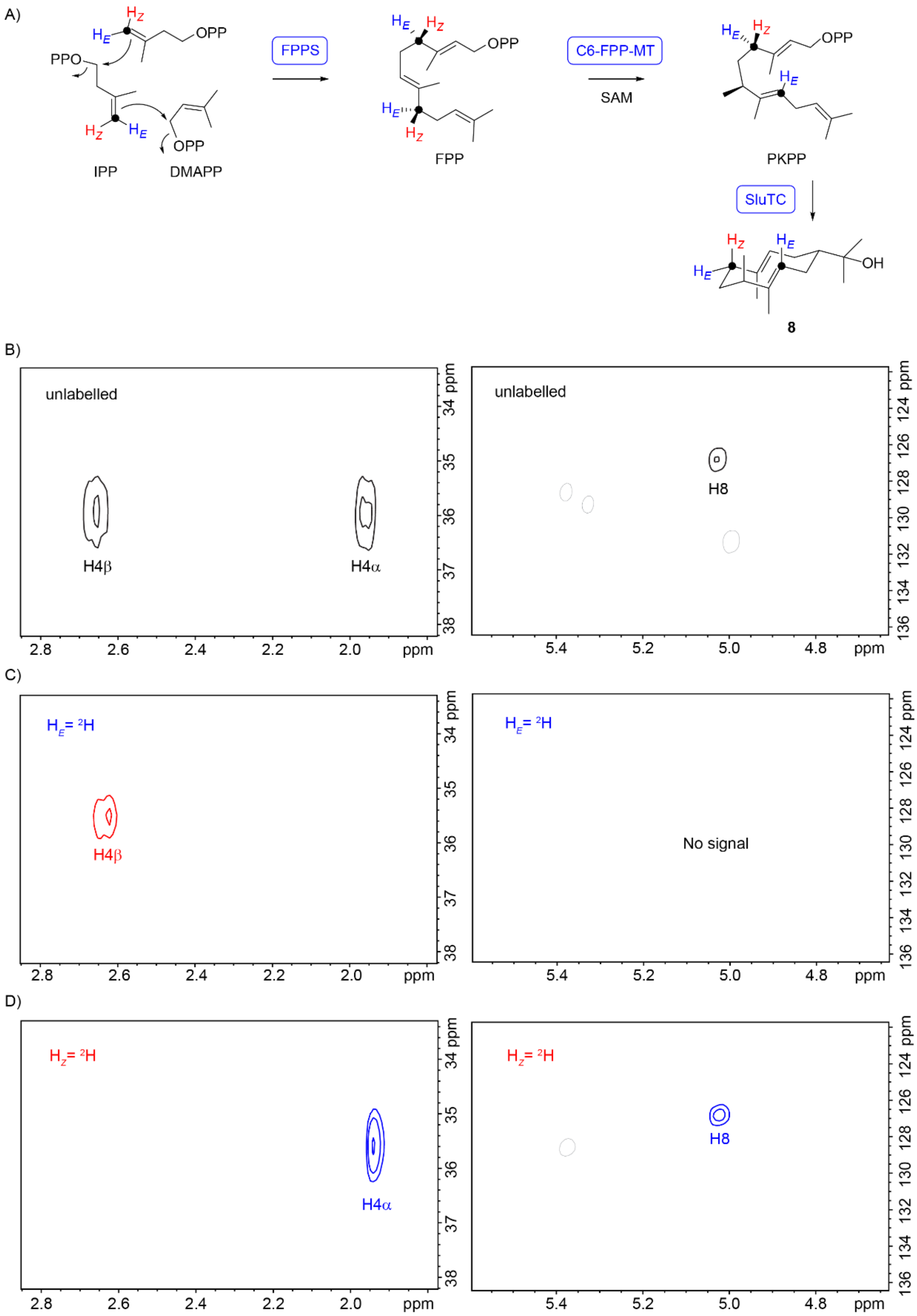

Figure S13. The absolute configuration of **8**. A) Conversion of (*R*)- and (*S*)-(1-^{13}C,1-^{2}H)IPP with IDI, FPPS, C6-FPP-MT and SluTC into labeled **8**. HSQC spectra of A) unlabeled **8** showing the region for C1, C5 and C9, C) labeled **8** obtained from (*R*)-(1-^{13}C,1-^{2}H)IPP (signals for H_S=^{1}H are observed), and D) labeled **8** obtained from (*S*)-(1-^{13}C,1-^{2}H)IPP (signals for H_R=^{1}H are observed). The results allow to assign the absolute configuration as shown in Scheme 1 of main text.

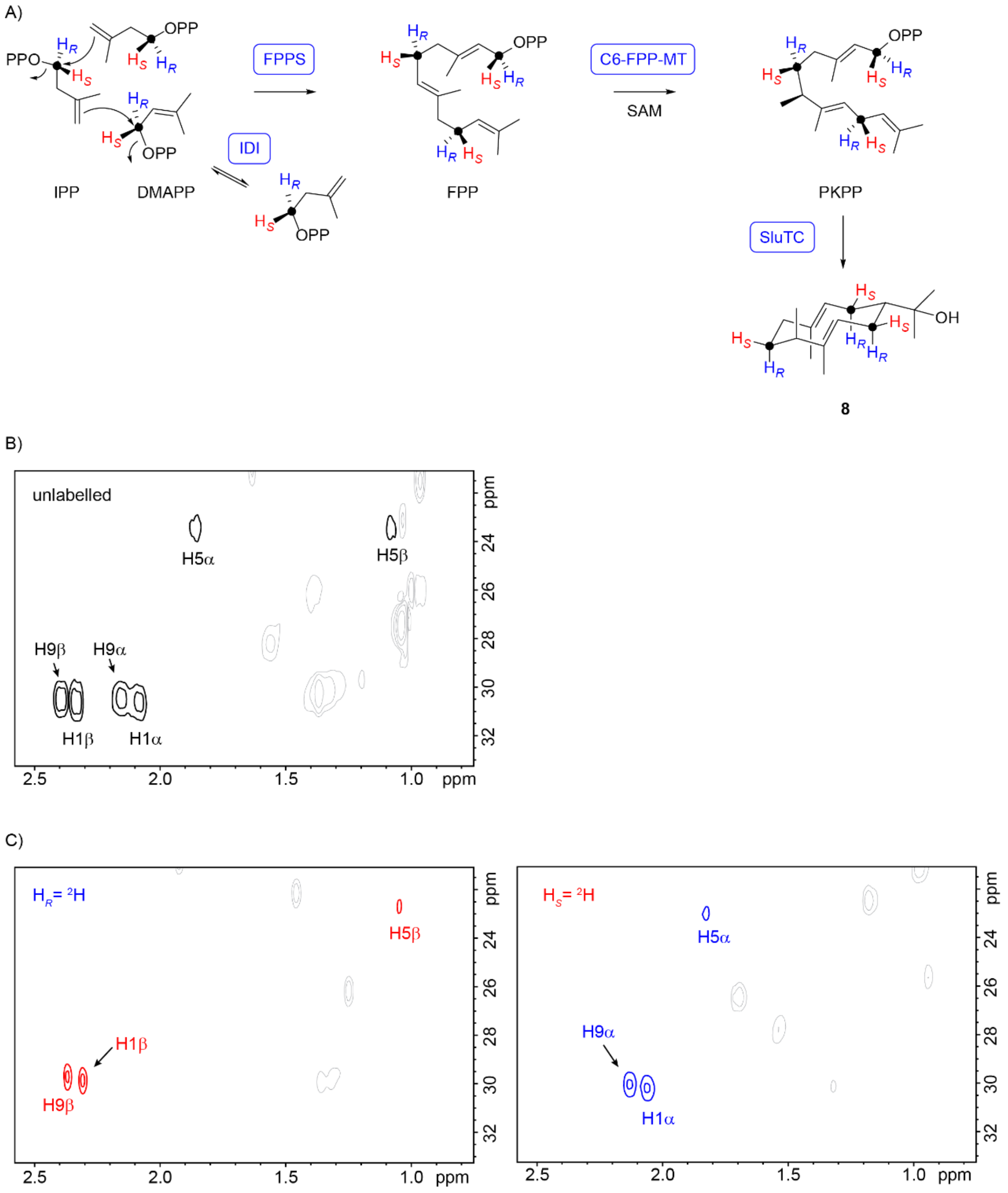

Figure S14. The absolute configuration of **8**. A) Conversion of DMAPP and (*E*)- and (*Z*)-(4-^{13}C,4-^{2}H)IPP with FPPS, C6-FPP-MT and SluTC into labeled **8**. HSQC spectra of A) unlabeled **8** showing the region for C4 and C8, C) labeled **8** obtained from (*E*)-(1-^{13}C,1-^{2}H)IPP (signals for H_Z=^{1}H are observed), and D) labeled **8** obtained from (*Z*)-(1-^{13}C,1-^{2}H)IPP (signals for H_E=^{1}H are observed). The results allow to assign the absolute configuration as shown in Scheme 1 of main text.

Table S2. Crystal data and structure refinement for **10**.

crystal habitus	clear colourless plank
device type	STOE STADIVARI
empirical formula	$C_{18}H_{35}O_{2.5}$
moiety formula	$C_{16}H_{30}O_2$, 0.5[$C_4H_{10}O$]
formula weight	291.46
temperature / K	100
crystal system	Monoclinic
space group	$P2_1$
a / Å	11.3383(9)
b / Å	14.3120(13)
c / Å	11.4938(9)
β / °	93.067(6)
volume / Å^3	1862.5(3)
Z	4
ρ_{calc} / g cm^{-3}	1.039
μ / mm^{-1}	0.516
F(000)	652
crystal size/mm^3	0.2 × 0.05 × 0.03
absorption correction	multi-scan
Tmin; Tmax	0.6050; 0.9670
radiation	Cu Kα (λ = 1.54186)
2Θ range for data collection / °	9.878 to 135.494°
completeness to Θ	1.005
index ranges	-10 ≤ h ≤ 13, -16 ≤ k ≤ 17, -12 ≤ l ≤ 13
reflections collected	24347
independent reflections	5952 [R_{int} = 0.1611, R_{sigma} = 0.0729]
data / restraints / parameters	5952/156/340
goodness-of-fit on F^2	0.941
final R indexes [I>=2σ (I)]	R_1 = 0.0917, wR_2 = 0.2405
final R indexes [all data]	R_1 = 0.1414, wR_2 = 0.2928
Largest diff. peak/hole / e Å^{-3}	0.27 / -0.29
Flack parameter	0.0(6)
Bijvoet-pair Bayesian statistics	P2(true) = 0.884; P3(true) = 0.270; P3(rac-twin) = 0.695; P3(false) = 0.035

X-ray crystallography

Clear colourless plank-like specimens of **10** have been obtained by recrystallisation from diethyl ether at 4 °C. The data collection was performed on a STOE StadiVari diffractometer using Cu-K_{α} (λ = 1.54178 Å) radiation. The diffractometer was equipped with a low-temperature device (Cryostream 800 series, Oxford Cryosystems, 100(2) K). Intensities were measured by fine-slicing φ- and ω-scans and corrected for background, polarization and Lorentz effects. A multi-scan absorption correction following Blessing's method was applied.[4] The structure was solved by intrinsic-phasing methods and refined anisotropically by the least-squares procedure implemented in the SHELX program system.[5] The hydrogen atoms were included isotropically using the riding model on the bound carbon atoms. The absolute configuration was determined by inspection of the Flack-parameter and by using Bayesian statistics on Bijvoet differences.[6,7]

The crystal quality only allowed for an uncertain determination of the absolute configuration. Since racemic twinning could be excluded the certainty of the correct absolute configuration according to Bijvoet-pair analysis is ca. 88%. The assigned absolute configuration is in agreement with the one determined through the stereoselective deuteration experiments.

CCDC number 2503478 contains the supplementary crystallographic data for this paper, which can be obtained free of charge from the Cambridge Crystallographic Data Centre via http://www.ccdc.cam.ac.uk/data_request/cif.

Enzyme crystallography and site-directed mutagenesis

Cloning and Protein Expression

The gene encoding the SAM-dependent C6-FPP methyltransferase from *Streptomyces varsoviensis* (WP_048831493) was obtained as a codon-optimized sequence for expression in *Escherichia coli* (Eurofins Genomics) and introduced into a modified pETDuet plasmid that produces an N-terminal His_6-SUMO fusion. DNA fragments were assembled using the NEBuilder HiFi cloning system according to the supplier's instructions. Mutations listed in Table S3 were generated with the Q5 site-directed mutagenesis kit (NEB), and all constructs were confirmed by Sanger sequencing (Eurofins Genomics). The plasmids were transformed into *E. coli* BL21 (DE3) cells and cultured in 2 L of LB medium containing 100 µg mL^{-1} ampicillin at 37 °C under shaking conditions. When the cultures reached an optical density of 0.6-0.8 at 600 nm, they were cooled on ice and protein expression was induced by adding 0.5 mM IPTG. Incubation proceeded overnight at 20 °C. The cells were harvested by centrifugation, washed with 0.9% NaCl, and stored frozen at –20 °C until purification.

Table S3. Primer sequences for mutagenesis with altered codons highlighted in bold.

Enzyme	Forward primer (5' → 3')	Reverse primer (5' → 3')
Δ15N	G GTG AAG CTA AAG TTC C	GGA TCC ACC GAT CTG
F45A	G CGT GTT CAC **GCG** CAC ATG GGT CTG	GGA CCC GGA CCG TAT
E165A	T GTT GCT TTC **GCG** TCT TCT GGT TAC ATG	GCA GCA CCG TAA GCA
E165Q	T GTT GCT TTC **CAG** TCT TCT GGT TAC ATG	GCA GCA CCG TAA GCA
H194A	T ATC CAG GAA **GCG** TTC CTG TGC CGT CCG	CCG AAC CAA CCA CCC
H194N	T ATC CAG GAA **AAC** TTC CTG TGC C	CCG AAC CAA CCA CCC
Y209A	C GAC GGT TAC **GCG** AAA ACC CGT CTG G	ATG AAA CGG GTC CAT TC
W244F	T GCT GAA TTC **TTT** GTT CAG TCT ATG G	GCA CGG TCG GTG ATG

Protein Purification

Frozen cell material (approximately 20 g) was resuspended in 50 mL buffer A (100 mM Tris-HCl, pH 7.5, 500 mM NaCl, 20 mM imidazole, 10 mM β-mercaptoethanol) and disrupted by ultrasonication using a Branson Digital Sonifier 250. After centrifugation at 40,000 × g for 30 min at 4 °C, the clarified lysate was loaded onto a HisTrap HP column (5 mL, GE Healthcare) equilibrated with buffer A and operated on an ÄKTA Pure system. The column was washed with buffer A containing 5% buffer B (buffer A supplemented with 500 mM imidazole), followed by elution with a linear gradient up to 100% buffer B over 50 mL. Protein-containing fractions were combined and treated with 0.5 mg SUMO protease (Ulp1) and dialyzed overnight at 4 °C against 2 L buffer C (20 mM Tris-HCl, pH 7.5, 100 mM NaCl, 2 mM β-mercaptoethanol). To remove residual tagged protein, the dialyzed material was passed over the same HisTrap column, and the flow-through was collected and concentrated to roughly 2 mL using Amicon Ultra-15 devices. After centrifugation at 20,000 × g for 10 min at 4 °C, the sample was applied to a HiLoad Superdex 200 16/60 column equilibrated with buffer D (buffer C containing 2 mM DTT and 5 mM $MgCl_2$). Fractions containing C6-FPP-MT were pooled, concentrated to at least 15 mg mL^{-1}, and either used directly or stored at –80 °C.

Protein Crystallization and Structure Determination

Crystals of C6-FPP-MT were generated by sitting-drop vapor diffusion at 4 °C. For ligand complexes, farnesyl pyrophosphate (FPP; 100 mM stock in water) and *S*-adenosylhomocysteine (SAH; 50 mM stock in dimethyl sulfoxide) were added to protein solutions at a final concentration of 2 mM per ligand using C6-FPP-MT at 10 mg mL^{-1}. Crystallization droplets contained up to 0.4 µL of protein and reservoir solution mixed in 1:1, 2:1, or 3:1 ratios. Drops were set using an ARI Crystal Gryphon pipetting system (Art Robbins Instruments). Crystals consistently formed in 0.1 M $MgCl_2$, 0.1 M 2-(N-morpholino)ethanesulfonic acid (MES, pH 6.5), and 30% polyethylene glycol 400. Suitable crystals appeared after several weeks at 4 °C. Individual crystals were vitrified directly from the mother liquor and diffraction data were recorded at beamline P13 (PETRA III, DESY, Hamburg, Germany; proposal MX-1019). Data were indexed, integrated, and scaled with XDS.[8] Details of data processing and refinement are listed in Table S4. Phasing was achieved by molecular replacement using an AlphaFold3-predicted model.[9] Model building was performed in COOT,[10] followed by restrained refinement in REFMAC5.[11] Water molecules were automatically added with ARP/wARP.[12] Final refinement included TLS parametrization, and the overall quality of the model was assessed with MOLPROBITY.[13] Criteria for the resolution cutoff included an average I/σ(I) above 2.0, an R_{merge} below 80% in the highest-resolution shell, and a redundancy of at least 3.0. The asymmetric unit contains twelve monomers of C6-FPP-MT bound to SAH, and a subset additionally accommodates the substrate FPP. Chain A was used for the ternary complex containing SAH and FPP, whereas chain B represents the SAH-bound state described in the manuscript. Coordinates and structure factors were deposited in the Protein Data Bank (accession code provided in Table S4).

Table S4. X-ray data collection and refinement statistics.

	C6-FPP-MT
**Crystal parameters**	
Space group	$P2_12_12_1$
Cell constants	a=130.6 Å
	b=133.3 Å
	c=231.4 Å
Subunits / AU[a]	12
**Data collection**	
Beam line	P13, DESY
Wavelength (Å)	0.976
Resolution range (Å)[b]	30-2.2 (2.3-2.2)
No. observations	776140
No. unique reflections[c]	201666
Completeness (%)[b]	98.7 / 99.6
R_{merge} (%)[b,d]	11.4 / 74.5
I/σ (I)[b]	7.5 / 2.0
**Refinement (REFMAC5)**	
Resolution range (Å)	30-2.2
No. refl. working set	191520
No. refl. test set	10080
No. non hydrogen	27119
No. of SAH atoms+	312
No. of FPP atoms	72
Solvent	1014
R_{work}/R_{free} (%)[e]	19.3 / 22.8
r.m.s.d. bond (Å) / (angle)[f]	0.002 / 0.7
Average B-factor ($Å^2$)	37.4
Ramachandran Plot (%)[g]	97.5 / 2.5 / 0
PDB accession code	**9TB4**

[a] Asymmetric unit; dataset has been collected from a single crystal.

[b] The values in parentheses for resolution range, completeness, R_{merge} and I/σ (I) correspond to the highest resolution shell.

[c] Data reduction was carried out with XDS and from a single crystal. Friedel pairs were treated as identical reflections.

[d] $R_{merge}(I) = \Sigma_{hkl}\Sigma_j \mid I(hkl)_j - \langle I(hkl)\rangle \mid / \Sigma_{hkl} \Sigma_j I(hkl)_j$, where $I(hkl)_j$ is the jth measurement of the intensity of reflection hkl and <I(hkl)> is the average intensity.

[e] $R = \Sigma_{hkl} \mid |F_{obs}| - |F_{calc}| \mid / \Sigma_{hkl} |F_{obs}|$, where R_{free} is calculated without a sigma cut off for a randomly chosen 5% of reflections, which were not used for structure refinement, and R_{work} is calculated for the remaining reflections.

[f] Deviations from ideal bond lengths/angles.

[g] Percentage of residues in favoured region / allowed region / outlier region.

Expression and purification of enzyme variants, incubation experiments

Small scale cultures of *E. coli* BL21 (DE3) harbouring pGro 7 plasmid transformed with the corresponding recombinant pYE-Express vectors containing the C6-FPP-MT variants were grown in LB medium (10 mL) amended with kanamycin sulfate and chloramphenicol overnight with shaking at 37 °C. Large scale expression cultures in LB medium (1 L) containing kanamycin sulfate and chloramphenicol were inoculated with the grown overnight culture (2 %). Culturing was continued with shaking at 37 °C until an OD_{600} = 0.4 – 0.6 was reached. After cooling the culture to 18 °C, enzyme expressions were induced by the addition of IPTG solution (100 mM, 1 ‰), and L-arabinose (1.3 M, 1 ‰) was used to induce the chaperones GroEL and GroES. The cultures were shaken at 18 °C for 18 h. Cells were harvested via centrifugation (1,500 g, 40 min, 4 °C), resuspended in binding buffer (20 mL, 4 °C) and lysed by ultrasonication (10x 1 min) on ice. The cell debris was removed by centrifugation (14,600 g, 10 min, 4 °C) and the supernatant was loaded on a Ni^{2+}-NTA superflow affinity chromatography column (Qiagen, Venlo, Netherlands) equilibrated with binding buffer. The column was washed with binding buffer (2 column volumes, CV, 4 °C) and washing buffer (2 CV, 4 °C). The desired proteins were eluted with elution buffer (1 CV, 4 °C). Protein concentrations were measured by Bradford assay.[2] All protein concentrations were adjusted to 1.0 mg mL^{-1} through dilution with incubation buffer for incubation experiments.

Enzymatic conversions of FPP (1 mg) plus *S*-adenosyl-L-methionine disulfate tosylate (2 mg) were performed in incubation buffer (1 mL) and C6-FPP-MT variants and SluTC were added at a concentration of 0.2 mg mL^{-1}. After incubation of 16 h at 30 °C, the reaction mixtures were extracted with n-hexane (200 µL), the extracts were dried with $MgSO_4$ and analysed by GC/MS. All experiments were performed in triplicates. Representative chromatograms for each enzyme variant are shown in Figure S15. The production of **8** and **10** by wild-type Sv-FPP-MT was set to 100%. Traces of products were observed for two enzyme variants (F45A: 0.01±0.001%, H194N: 0.04±0.01%, relative to wild-type production), while for all other enzyme variants the production was below the limits of detection (<0.01%).

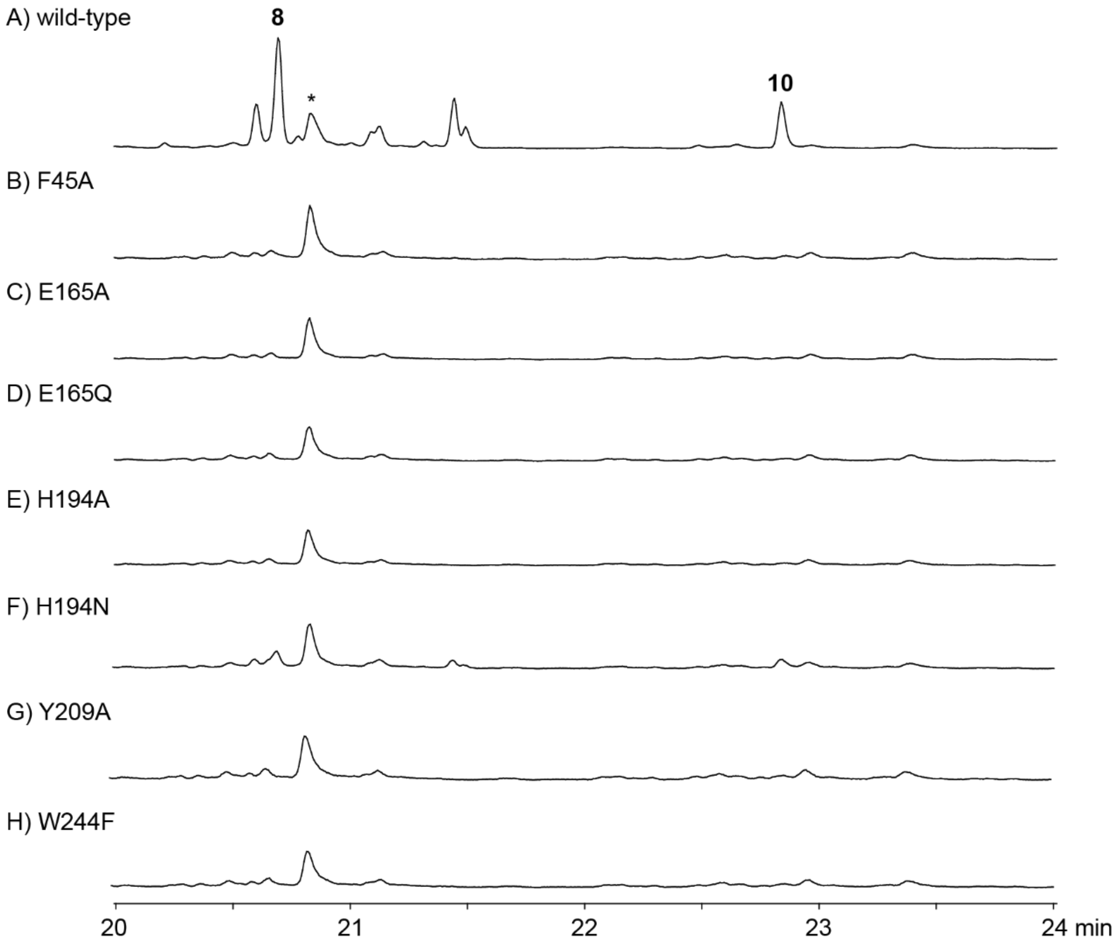

Figure S15. Total ion chromatograms of extracts obtained from enzyme reactions of FPP and SAM with Sv-FPP-MT (wild-type and its enzyme variants as indicated at each chromatogram) and SluTC. Only with wild-type Sv-FPP-MT the production of **8** and **10** was observed, while for all enzyme variants no product formation was found. The asterisk indicates a spontaneous decomposition product from FPP.

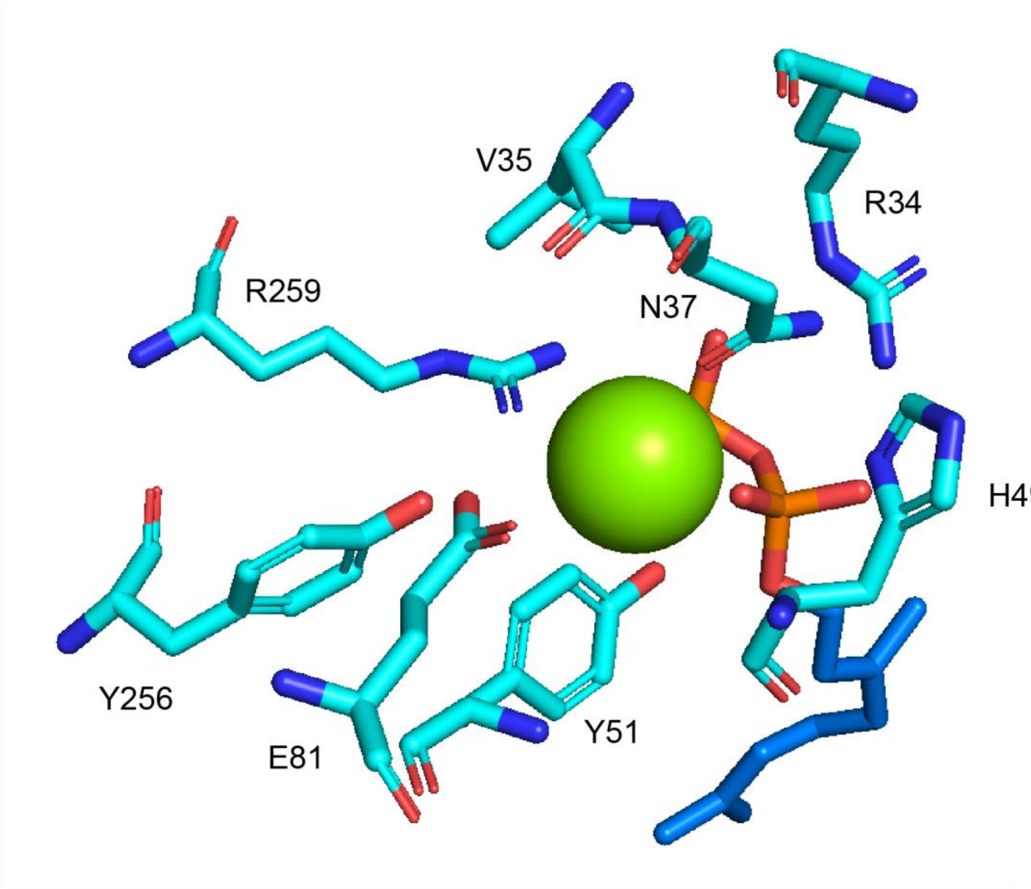

Figure S16. Residues involved in Mg^{2+} binding and interaction with the pyrophosphate group of the substrate GPP in C2-GPP-MT (3VC2). Mg^{2+} is shown as green sphere, the hydrocarbon chain of GPP is shown in blue.

A)

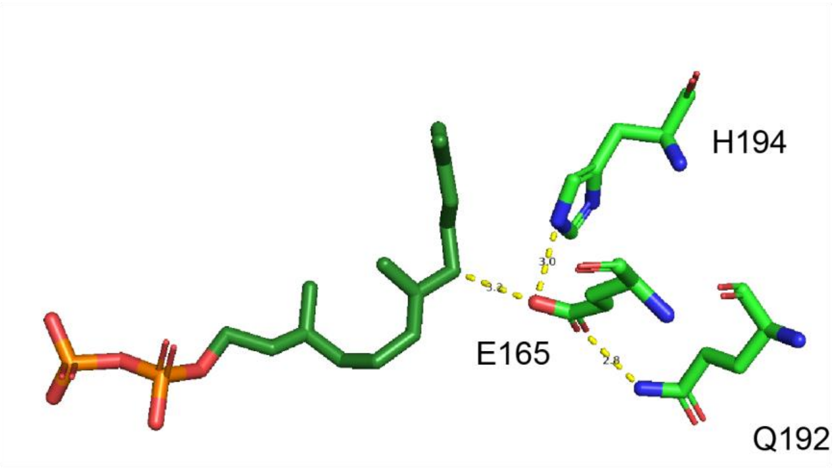

B)

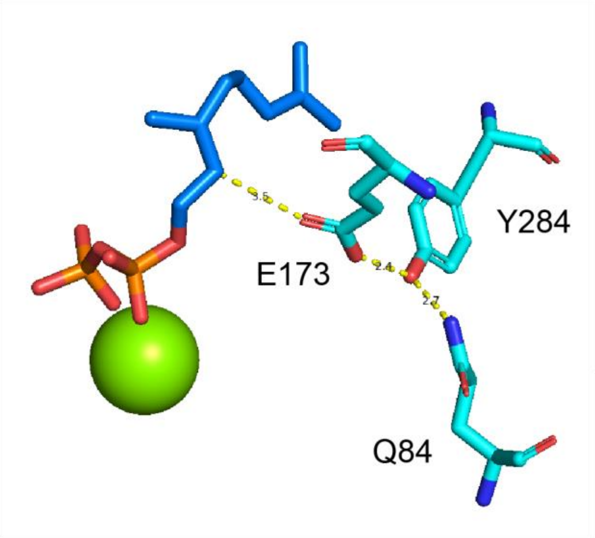

Figure S17. The conserved Brønsted base Glu165. A) In C6-FPP-MT Glu165 shows a distance of 3.2 Å to C8 of FPP and is engaged in hydrogen bonds to Gln192 and His194. B) In C2-GPP-MT the analogous residue Glu173 shows a distance of 3.5 Å to C2 and is involved in a hydrogen bonding network with Tyr284 and Gln84.

Table S5. Conservation of active site residues in C6-FPP-MT and 501 closely related homologs.

position[a]	residues found in the alignment
K36	**K: 99.8%**, R: 0.2%
Y37	**Y: 99.8%**, Q: 0.2%
F45	**F: 97.6%**, Y: 1.2%
M47	V: 66.1%, M: 26.1%, L: 7.2%, I: 0.4%, A: 0.2%
E165	**E: 100%**
Y169	**Y: 97.0%**, H: 3.0%
Q192	**Q: 97.2%**, E: 2.4%
H194	**H: 99.8%**, V: 0.2%
I205	I: 66.9%, M: 17.7%, V: 11.4%, L: 3.8%, F: 0.2%
Y209	**Y: 99.8%**, W: 0.2%
T211	**T: 100%**
F243	**F: 100%**
W244	**W: 100%**
H279	**H: 99.6%**, Q: 0.4%

[a] Position in C6-FPP-MT from *Streptomyces varsoviensis*.

References

[1] T. Reuter, L. Dieminger, S. Steidle, K. Zoller, M. Holocher, L. Zhou, D. Hanauska, K. Racz, L. Barra, Non-canonical C_{16} Homoterpene Biosynthesis Widespread in Actinobacteria, *Angew. Chem. Int. Ed.* **2024**, *63*, e202418613; *Angew. Chem.* **2024**, *136*, e202418613.

[2] M. M. Bradford, A rapid and sensitive method for the quantitation of microgram quantities of protein utilizing the principle of protein-dye binding. *Anal. Biochem.* **1976**, *72*, 248–254.

[3] G. R. Fulmer, A. J. M. Miller, N. H. Sherden, H. E. Gottlieb, A. Nudelman, B. M. Stoltz, J. E. Bercaw, K. I. Goldberg, NMR Chemical Shifts of Trace Impurities: Common Laboratory Solvents, Organics, and Gases in Deuterated Solvents Relevant to the Organometallic Chemist. *Organometallics* **2010**, *29*, 2176–2179.

[4] R. H. Blessing, An empirical correction for absorption anisotropy. *Acta Cryst. A* **1995**, *51*, 33–38.

[5] G. M. Sheldrick, SHELXT – Integrated space-group and crystal-structure determination. *Acta Cryst. A* **2015**, *71*, 3–8.

[6] H. D. Flack, On enantiomorph-polarity estimation. *Acta Cryst. A* **1983**, 39, 876–881.

[7] R. W. W. Hooft, K. L. H. Straver, A. L. Spek, Determination of absolute structure using Bayesian statistics on Bijvoet differences. *J. Appl. Cryst.* **2008**, *41*, 96–103.

[8] W. Kabsch, XDS. Acta Cryst. **2010**, *66D*, 125–132.

[9] J. Abramson, J. Adler, J. Dunger, R. Evans, T. Green, A. Pritzel, O. Ronneberger, L. Willmore, A. J. Ballard, J. Bambrick, S. W. Bodenstein, D. A. Evans, C.-C. Hung, M. O'Neill, D. Reiman, K. Tunyasuvunakool, Z. Wu, A. Zemgulyte, E. Arvaniti, C. Beattie, O. Bertolli, A. Bridgland, A. Cherepanov, M. Congreve, A. I. Cowen-Rivers, A. Cowie, M. Figurnov, F. B. Fuchs, H. Gladman, R. Jain, Y. A. Khan, C. M. R. Low, K. Perlin, A. Potapenko, P. Savy, S. Singh, A. Stecula, A. Thillaisundaram, C. Tong, S. Yakneen, E. D. Zhong, M. Zielinski, A. Zidek, V. Bapst, P. Kohli, M. Jaderberg, D. Hassabis, J. M. Jumper, Accurate structure prediction of biomolecular interactions with AlphaFold 3. *Nature* **2024**, *630*, 493–500.

[10] P. Emsley, B. Lohkamp, W. G. Scott, K. Cowtan, Features and development of Coot. *Acta Cryst.* **2010**, *66D*, 486–501.

[11] G. N. Murshudov, P. Skubák, A. A. Lebedev, N. S. Pannu, R. A. Steiner, R. A. Nicholls, M. D. Winn, F. Long, A. A. Vagin, REFMAC5 for the refinement of macromolecular crystal structures. Acta Cryst. **2011**, *67D*, 355–367.

[12] R. J. Morris, A. Perrakis, V. S. Lamzin, ARP/wARP and automatic interpretation of protein electron density maps. *Meth. Enzymol.* **2003**, *374*, 229–244.

[13] C. J. Williams, J. J. Headd, N. W. Moriarty, M. G. Prisant, L. L. Videau, L. N. Deis, V. Verma, D. A. Keedy, B. J. Hintze, V. B. Chen, S. Jain, S. M. Lewis, W. B. Arendall, J. Snoeyink, P. D. Adams, S. C. Lovell, J. S. Richardson, D. C. Richardson, MolProbity: More and better reference data for improved all-atom structure validation. *Prot. Sci.* **2018**, *27*, 293–315.
